# PRDX4 expression potentially links redox adaptation to oncogenic signaling and tumor progression in pancreatic ductal adenocarcinoma

**DOI:** 10.1016/j.tranon.2026.102865

**Published:** 2026-06-20

**Authors:** Yao Liu, Jia Han, Akihiro Shioya, Takeru Oyama, Xin Guo, Qian Yang, Hidetaka Uramoto, Sohsuke Yamada

**Affiliations:** a Department of Pathology and Laboratory Medicine, Kanazawa Medical University, Ishikawa 920-0293, Japan; b Department of Pathology, the Fourth Hospital of Hebei Medical University, Shijiazhuang, Hebei Province 050011, China; c Department of Pathology, Kanazawa Medical University Hospital, Ishikawa 920-0293, Japan; d Research Center, First Affiliated Hospital of Hebei University of Chinese Medicine, Shijiazhuang, Hebei Province 050011, China; e Department of Spleen and Stomach Diseases, First Affiliated Hospital of Hebei University of Chinese Medicine, Shijiazhuang, Hebei Province 050011, China; f Hebei Key Laboratory of Turbidity Toxin Syndrome, First Affiliated Hospital of Hebei University of Chinese Medicine, Shijiazhuang, Hebei Province 050011, China; g Second Department of Surgery, School of Medicine, University of Occupational and Environmental Health, Kitakyushu 807-8555, Japan; h Department of Thoracic Surgery, Kanazawa Medical University Hospital, Ishikawa 920-0293, Japan

**Keywords:** Pancreatic ductal adenocarcinoma (PDAC), Peroxiredoxin 4 (PRDX4), Redox homeostasis, NRF2/HO-1, Chemoresistance

## Abstract

**Background:**

Pancreatic ductal adenocarcinoma (PDAC) is characterized by aggressive behavior, therapeutic resistance, and redox imbalance. Peroxiredoxin 4 (PRDX4), an endoplasmic reticulum-localized antioxidant enzyme, has been implicated in tumor progression, but its clinical significance and redox-associated role in PDAC remain unclear.

**Methods:**

PRDX4 expression was assessed by immunohistochemistry in 128 resected PDAC specimens and correlated with clinicopathological features and disease-specific survival (DSS). Functional effects were examined in PRDX4-overexpressing PDAC cell lines. Intracellular reactive oxygen species (ROS) were evaluated under basal and oxidative stress conditions, with N-acetyl-l-cysteine (NAC) rescue. Signaling and redox-related proteins were analyzed by Western blotting. A PRDX4-transgenic mouse model was used to evaluate tumor growth, angiogenesis, and gemcitabine (GEM) responsiveness.

**Results:**

High PRDX4 expression was associated with aggressive clinicopathological features and poor DSS. PRDX4 overexpression promoted cell proliferation and migration and reduced intracellular ROS levels. NAC treatment diminished ROS differences, supporting ROS-dependent redox modulation. PRDX4 overexpression was associated with increased ERK phosphorylation, suppressed JNK phosphorylation, increased total PI3K/AKT and p38 levels, reduced E-cadherin expression, and upregulation of NRF2 and HO-1. PRDX4 expression positively correlated with NRF2 and HO-1 in clinical specimens. In vivo, PRDX4 overexpression promoted tumor growth, increased microvessel density, and reduced GEM sensitivity.

**Conclusions:**

PRDX4 is associated with aggressive tumor behavior, redox adaptation, and might reduce gemcitabine responsiveness in PDAC in vivo, supporting its potential as a prognostic biomarker and a candidate molecule for future therapeutic investigation.

## Highlights

• High PRDX4 expression is associated with aggressive clinicopathological features and poor disease-specific survival in PDAC.
• PRDX4 overexpression promotes PDAC cell proliferation, migration, and redox-adaptive capacity.
• PRDX4 reduces intracellular ROS accumulation and is associated with altered MAPK- and PI3K/AKT-related signaling.
• PRDX4 overexpression is associated with enhanced tumor growth, angiogenesis, and reduced gemcitabine responsiveness in vivo.
• PRDX4 may serve as a prognostic biomarker and redox-associated therapeutic target in PDAC.

## Introduction

Pancreatic ductal adenocarcinoma (PDAC) is one of the most aggressive and lethal malignancies worldwide. Globally, PDAC ranks among the top causes of cancer-related mortality, with an estimated annual incidence of over 495,773 new cases and 466,003 deaths, reflecting its extremely poor prognosis and limited therapeutic options [1,2]. Due to its aggressive biological behavior, early metastatic potential, and resistance to conventional therapies, the five-year overall survival rate for PDAC remains below 10.8% [3]. While surgical resection offers the only potential for a cure, the majority of patients present with advanced disease at the time of diagnosis, and postoperative recurrence rates are high [4]. Despite the widespread clinical use of GEM-based regimens, the survival benefit remains modest, underscoring the urgent need to elucidate the core molecular mechanisms driving PDAC aggressiveness, immune evasion, and therapeutic resistance [5].

A hallmark of PDAC is its unique tumor microenvironment (TME), characterized by dense desmoplasia, hypoxia, and a profound exclusion of effective anti-tumor immunity [6,7]. Increasing evidence suggests that oxidative stress and redox imbalance are central to pancreatic oncogenesis [8]. Driven by oncogenic KRAS signaling, mitochondrial dysfunction, and stromal-inflammatory crosstalk, PDAC cells continuously generate high levels of reactive oxygen species (ROS) [9]. To maintain oncogenic signaling while evading oxidative damage, cancer cells undergo "redox reprogramming" by activating robust antioxidant systems that support proliferation, invasion, and therapeutic resistance [10].

Among the intracellular antioxidant defenses, the peroxiredoxin (PRDX) family plays a pivotal role in regulating hydrogen peroxide (H_2_O_2_) signaling and maintaining redox homeostasis [11]. Peroxiredoxin 4 (PRDX4) is the only member of the peroxiredoxin family that is predominantly localized to the endoplasmic reticulum (ER) and the secretory pathway. Within the ER, PRDX4 functions as a key redox enzyme that utilizes H_2_O_2_ generated by endoplasmic reticulum oxidoreductin-1 (ERO1) to catalyze the oxidation of protein disulfide isomerase (PDI), thereby promoting disulfide bond formation during oxidative protein folding and maintaining ER proteostasis [12]. This unique subcellular localization distinguishes PRDX4 from other PRDX family members and places it at a critical interface between intracellular redox regulation, protein secretion, and TME remodeling. PRDX4 has also been implicated in the regulation of metabolic and inflammatory disorders across multiple organ systems [13]. Recent studies have highlighted the importance of redox adaptation in supporting PDAC progression and therapeutic resistance [10]. Although PRDX4 has been implicated in redox regulation and oxidative protein folding within the ER, direct experimental evidence linking PRDX4 expression to intracellular ROS modulation in PDAC cells remains limited. Therefore, direct measurement and functional validation of intracellular ROS levels are necessary to better define the redox-associated biological role of PRDX4 in PDAC. Recently, aberrant PRDX4 expression has been identified in various cancers, including gastric [14], colorectal [15], and breast cancers [16], and has been linked to epithelial-mesenchymal transition (EMT), high invasiveness, and poor prognosis [17,18]. Our previous studies have also demonstrated that PRDX4 exerts complex regulatory roles in the progression of hepatocellular carcinoma and lung cancer [19,20]. In PDAC, PRDX4 expression levels have been identified as a significant predictor of clinical outcomes, suggesting its biological function may be highly dependent on specific redox demands and microenvironmental cues [21].

Recent studies indicate that PRDX4 may function as a redox-sensitive signaling hub, modulating key oncogenic cascades including the MAPK and PI3K/AKT pathways [22]. However, given that PRDX4 is predominantly localized within the ER or secretory pathway, its impact on cytosolic signaling is unlikely to result from direct enzymatic activity in the cytoplasm. Instead, such effects may be mediated through indirect mechanisms, including modulation of ER redox balance, protein maturation, or secretory pathway activity; however, these possibilities remain speculative and were not directly examined in the present study. In particular, whether PRDX4 overexpression influences ER stress responses or activates the unfolded protein response (UPR) was not assessed in this study and warrants further investigation using established ER stress markers. Due to its unique ER localization, PRDX4 is ideally positioned to regulate the maturation and secretion of proteins that remodel the structure and function of the TME [23]. This link between intracellular redox adaptation and extracellular microenvironment remodeling likely underpins the immunosuppressive landscape and chemoresistance observed in PDAC [24]. In addition to redox regulation and immune modulation, angiogenesis is a key component of tumor microenvironment remodeling in PDAC [25]. Although PDAC is typically hypovascular, emerging evidence suggests that aberrant neovascularization contributes to tumor progression, chemoresistance, and drug delivery [26]. Redox balance and ROS signaling are closely involved in angiogenesis, as ROS can regulate endothelial cell behavior and vascular remodeling through redox-sensitive pathways such as MAPK and PI3K/AKT [27]. Given its role in maintaining intracellular redox homeostasis and its localization within the ER and secretory pathway, PRDX4 may influence the maturation or secretion of pro-angiogenic factors, thereby contributing to vascular remodeling. However, the relationship between PRDX4 expression and angiogenesis in PDAC remains unclear, and the integrated role of PRDX4 in coordinating redox signaling, angiogenic remodeling, and chemotherapeutic response in PDAC has not been systematically investigated.

While our previous study identified PRDX4 as a prognostic biomarker in PDAC, the present study extends this work by investigating the role of PRDX4 within a broader biological context, with a particular focus on redox regulation, tumor progression, and therapeutic response. We evaluated the clinical and biological significance of PRDX4 expression in PDAC using 128 surgically resected specimens, analyzing its association with clinicopathological features and patient survival. The in vitro experiments examined the effects of PRDX4 overexpression on cell proliferation, migration, ROS modulation, and redox- and signaling-related protein expression. Furthermore, using a PRDX4-transgenic mouse model, we assessed tumor growth, angiogenesis, and GEM responsiveness in vivo. Collectively, this study provides a more comprehensive understanding of the redox-associated role of PRDX4 in PDAC progression and may offer a rationale for future redox-targeted therapeutic strategies.

## Materials and methods

### Tissue samples

This study utilized surgical specimens from 128 patients with PDAC who underwent surgical resection at multiple centers (Kanazawa Medical University Hospital, Independent Administrative Agency National Hospital Organization Fukuyama Medical Center, and Kagoshima University Hospital) in Japan between 2005 and 2017. A small subset of patients underwent palliative tumor resection despite the presence of distant metastasis (pM1), based on intraoperative findings or symptom-oriented surgical decision-making. Inclusion criteria were as follows: (1) histologically confirmed PDAC; (2) availability of formalin-fixed, paraffin-embedded (FFPE) tumor tissue suitable for immunohistochemical analysis; and (3) complete clinicopathological and follow-up data. Both curative-intent resections and selected palliative resections were included, reflecting real-world surgical practice during the study period. Exclusion criteria comprised: (1) perioperative mortality, defined as death during the initial hospitalization or within 30 days after surgery; (2) presence of concurrent malignant tumors; and (3) severe comorbidities that markedly limited life expectancy. Notably, none of the included patients received systemic neoadjuvant therapy, including chemotherapy or radiotherapy, prior to surgical resection. All tissue samples were therefore obtained from treatment-naïve primary tumors, eliminating potential confounding effects of preoperative systemic therapy on PRDX4 expression. Histopathological evaluation was performed by three board-certified pathologists (Y.L., T.O., S.Y.), with unanimous consensus achieved in diagnostic assessments. Histological classification adhered to the fifth edition of the WHO Classification of Digestive System Tumors.

DSS was defined as the interval from diagnosis/treatment initiation to death directly attributable to PDAC, excluding unrelated causes (e.g., accidents, non-cancer diseases). Postoperative follow-up included monthly clinical evaluations during the first year and bimonthly to trimonthly assessments thereafter, incorporating abdominal computed tomography (CT), magnetic resonance imaging (MRI), and carcinoembryonic antigen measurements. CT/MRI surveillance was conducted semiannually for three years post-surgery. Metastatic lesions were managed via surgical resection and/or chemotherapy. Suspected recurrences prompted immediate diagnostic investigations. This study was conducted in accordance with the Declaration of Helsinki and approved by the Ethics Committee of Kanazawa Medical University (Approval No I233). Informed consent was obtained from all participants.

### Histopathological evaluation and immunohistochemistry (IHC)

FFPE tissues from 128 PDAC cases were sectioned at 4 μm thickness and subjected to hematoxylin and eosin (H&E) staining for histopathological confirmation. IHC staining for PRDX4 (Thermo Fisher Scientific, #PA5–85252, 1:1000) was performed using the Histofine Simple Stain MAX-PO kit (Nichirei Biosciences, Japan). IHC staining for NRF2 (CST, #12721, 1:500) and HO-1 (CST, #26416, 1:500) was performed using the Histofine Simple Stain MAX-PO kit (Nichirei Biosciences, Japan). After deparaffinization and rehydration, antigen retrieval was carried out by trypsin digestion, followed by blocking of endogenous peroxidase activity with 0.5% hydrogen peroxide for 15 min at room temperature. Sections were incubated with the primary antibody overnight at 4 °C and subsequently treated with the secondary antibody for 30 min at room temperature. Chromogenic visualization was achieved using 3,3′-diaminobenzidine (DAB), and nuclei were counterstained with hematoxylin. Positive and negative control slides were included in each staining batch to ensure quality consistency. The expression level of PRDX4, NRF2 and HO-1 were quantified as the percentage of positively stained tumor cells. Only cytoplasmic staining in tumor cells was considered positive for PRDX4 and HO-1. NRF2 tumor cells were considered positive for both cytoplasmic and nuclear staining. Staining intensity was relatively homogeneous across tumor cells in most cases, with minimal variability compared with the proportion of positive cells. Therefore, a percentage-based scoring method was adopted for semi-quantitative evaluation to improve reproducibility and reduce subjective bias associated with intensity grading. The PRDX4 expression cut-off value (13.5%) was determined by ROC analysis (fig. S1) and further evaluated by sensitivity analyses using alternative thresholds (median- and tertile-based), which yielded consistent prognostic trends (Table S1). To further assess the robustness of the ROC-derived cut-off value, a bootstrap resampling approach was performed. The dataset was resampled with replacement for 2000 iterations, and in each bootstrap sample, the optimal cut-off value was recalculated using the Youden index. The distribution of bootstrap-derived cut-off values was examined to evaluate the stability of the selected threshold. All slides were digitized using the NanoZoomer Digital Pathology Virtual Slide Viewer (Hamamatsu Photonics, Japan) for quantitative assessment. Three independent board-certified pathologists (Y.L., T.O., S.Y.), blinded to clinicopathological and survival data, evaluated all slides. Inter-observer agreement exceeded substantial, and discrepant cases were reviewed jointly to reach a consensus (Table S2).

### Cell lines and plasmid transfection

Human pancreatic cancer cell lines (MiaPaCa-2, AsPC-1, and PANC-1) were obtained from American Type Culture Collection (ATCC), CRL-11268, CRL-1682, CRL-1469, respectively, and cultured in DMEM and RPMI-1640 supplemented with 10% fetal bovine serum and antibiotics at 37 °C in a humidified atmosphere with 5% CO_2_. MiaPaCa-2, AsPC-1, and PANC-1 pancreatic cancer cell lines represent distinct biological phenotypes of PDAC. MiaPaCa-2 cells exhibit a poorly differentiated, highly glycolytic phenotype with pronounced proliferative capacity; AsPC-1 cells are characterized by strong invasive potential and metastatic origin; whereas PANC-1 cells display an intermediate phenotype with partial epithelial–mesenchymal plasticity. The use of these three cell lines allowed evaluation of PRDX4 function across heterogeneous PDAC cellular contexts. Cells were transfected with PRDX4 overexpressing plasmid DNA or empty vector using Lipofectamine® 2000 and Opti-MEM medium (Life Technologies, Carlsbad, CA, USA) according to the manufacturer’s instructions. pMCV-Tag-2b (a gift from the Department of Pathology, Kagoshima University) vectors were used as a negative control. Transfection efficiency was confirmed by Western blotting.

### Cell proliferation and migration assays

Cell proliferation was assessed using the Cell Counting Kit-8 (CCK-8, Dojindo). Transfected cells were seeded into 96-well plates at a density of 5 × 10^3^ cells per well and incubated for 24, 48, and 72 h. Each condition was performed in triplicate wells. Absorbance was measured at 450 nm using a microplate reader. For migration assays, transwell chambers (Catalog Number: 140675, Thermo Fisher Scientific) were used. Transfected cells (5 × 10^4^ per well) were seeded in serum-free medium in the upper chamber, while medium containing 10% FBS was placed in the lower chamber. After 24 h, migrated cells on the bottom surface of the membrane were fixed, stained with 4′,6-diamidino-2-phenylindole (DAPI), and counted in five randomly selected HPFs.

### Measurement of intracellular reactive oxygen species (ROS)

Intracellular ROS levels were measured using a fluorescence probe-based ROS detection assay according to the manufacturer’s instructions. The ROS detection kit (Highly Sensitive DCFH-DA, code R252, Dojindo Laboratories, Kumamoto, Japan) was used in this study. Representative PANC-1 cells were seeded into 6-well plates and allowed to adhere overnight. For antioxidant treatment, N-acetyl-l-cysteine (NAC; Sigma-Aldrich, St. Louis, MO, USA) was freshly dissolved in sterile PBS to prepare a stock solution and diluted to the indicated working concentration in culture medium immediately before use. For oxidative stress induction, cells were treated with hydrogen peroxide (H_2_O_2_; 30% stock solution, FUJIFILM Wako Pure Chemical Corporation, Osaka, Japan) at a final concentration of 200 μM for 30 min prior to ROS detection. For combined treatment experiments, cells were pretreated with NAC for 1 h before H_2_O_2_ exposure. After the indicated treatments, cells were incubated with 2′,7′-dichlorodihydrofluorescein diacetate (DCFH-DA) working solution at 37 °C for 30 min in the dark. Following incubation, cells were washed three times with serum-free medium to remove excess probe. Fluorescence images were captured using a fluorescence microscope under identical acquisition and exposure settings across all experimental groups. Fluorescence intensity was quantified using image analysis software or a fluorescence microplate reader (Ex/Em = 488/525 nm), as appropriate. For quantitative analysis, mean fluorescence intensity (MFI) was calculated from at least five randomly selected fields per sample. ROS levels were normalized to cell number to minimize potential bias related to cell density differences. All experiments were performed in at least three independent biological replicates.

### Western blot analysis

Whole cell lysates were prepared using RIPA buffer (Thermo Fisher Scientific) supplemented with protease and phosphatase inhibitors. Protein concentrations were determined by the BCA assay (Thermo Fisher Scientific). Equal amounts of protein (30–50 μg) were separated by SDS-PAGE and transferred onto PVDF membranes (Millipore). After blocking with 5% skim milk for 1 h at room temperature, membranes were incubated overnight at 4 °C with the following primary antibodies: PRDX4 (1:1000, Thermo Fisher, #PA5–85252), ERK1/2 (1:1000, CST, #4695), p-ERK (1:1000, CST, #4370), JNK (1:1000, CST, #9252), p-JNK (1:1000, CST, #4668), p38 MAPK (1:1000, CST, #8690), p-p38 MAPK (1:1000, CST, #4511), PI3K p85 (1:500, CST, #4257), p-PI3K p85 (1:500, CST, #17366), AKT (1:1000, CST, #9272), p-AKT (1:1000, CST, #4060), Cyclin D1 (1:500, CST, #2978), E-cadherin (1:500, CST, #3195), NRF2 (1:500, CST, #12721), HO-1 (1:500, CST, #26416), β-actin (1:1000, CST, #4970). Following incubation with HRP-conjugated anti-rabbit IgG secondary antibody (1:1000, CST, #7074) for 1 h at room temperature, protein bands were visualized using enhanced chemiluminescence (ECL; Bio-Rad) and imaged with a ChemiDoc detection system. Densitometric quantification was performed using Image J software. All Western blot experiments were conducted using at least three independent biological replicates. Densitometric analysis was performed using data from all independent experiments, with protein expression levels normalized to β-actin. Western blot analyses were performed in multiple PDAC cell lines. Representative results are shown for PANC-1 cells, which were selected as a primary model due to their robust transfection efficiency, stable PRDX4 overexpression, and consistent functional responses. To further confirm the reproducibility of key findings across different cellular contexts, selected signaling- and redox-related proteins were additionally validated in MiaPaCa-2 cells.

### Quantitative real-time PCR (RT-qPCR)

Quantitative real-time polymerase chain reaction (RT-qPCR) was performed in PANC-1 cells to evaluate mRNA expression levels. Total RNA was extracted using the ReliaPrep™ RNA Tissue Miniprep kit (Promega, Madison, WI, USA) according to the manufacturer’s instructions. Reverse transcription was carried out using PrimeScript™ RT Master Mix (TaKaRa, Kyoto, Japan). Quantitative PCR was performed using TB Green™ Premix Ex Taq™ (TaKaRa, Kyoto, Japan) on a real-time PCR system. The relative mRNA expression levels were normalized to 18S and calculated using the 2^−ΔCT^ method. The primers were synthesized by Life Technologies, and the primer sequences are showed in Table S3.

### Animal models and in vivo experiments

PRDX4 Tg mice were generated as previously described [28]. C57BL/6 mice were purchased from Charles River Laboratories (Yokohama, Japan) as wild type (WT). Eight to ten weeks old male C57BL/6 WT mice and PRDX4 transgenic (Tg) mice were used for in vivo studies. Male mice were selected to reduce variability related to estrous cycle–associated hormonal fluctuations in exploratory mechanistic experiments. The sample size was determined based on exploratory mechanistic objectives and prior similar studies, and all analyses were interpreted accordingly. Animals were maintained under specific pathogen-free (SPF) conditions in a temperature and humidity-controlled facility with a 12-hour light/dark cycle and ad libitum access to food and water. To establish syngeneic subcutaneous tumor model, Pan02 murine pancreatic cancer cells (5 × 10^6^ in 100μl PBS) were injected subcutaneously into the dorsal flank of WT and Tg mice. Tumor growth was monitored weekly using digital calipers, and tumor volume was calculated using the formula: Volume = 1/2×length×(width)^2^. When tumors reached approximately 150–200 mm^3^, mice were randomized into treatment groups (n = 4 per group) receiving either saline (control) or GEM (50 mg/kg/week, intraperitoneally) for 4 consecutive weeks. There was no significant difference in tumor volume between WT and Tg mice at the initiation of GEM treatment, ensuring comparable baseline tumor burden across genotypes. Body weight and overall health status were recorded throughout the study. At the experimental endpoint, mice were sacrificed, and tumors were excised, measured, and processed for histopathological examination. The tumors were excised for H&E and IHC (PRDX4 [1:1000, Thermo Fisher, #PA5–85252], CD34 [1:500, CST, #71771], NRF2 [1:500, CST, #12721], HO-1 [1:500, CST, #26416]) analysis. After deparaffinization and antigen retrieval, sections were incubated with primary antibodies against CD34, NRF2, and HO-1, followed by appropriate secondary antibodies and DAB visualization. The extent of PRDX4, microvessel density (MVD), NRF2 and HO-1 were semi-quantitatively assessed by three blinded pathologists (Y.L., T.O., S.Y.). For NRF2, both nuclear and cytoplasmic staining were recorded, whereas HO-1 expression was mainly evaluated in the cytoplasm.

### Genotyping of PRDX4-transgenic mice

Genomic DNA was extracted from 2–3 mm mouse tail biopsies using the KAPA Mouse Genotyping Kit (KK7153, KAPA Biosystems) following the manufacturer’s protocol. Briefly, each tail sample was incubated in a 100 μL lysis mixture containing 88 μL KAPA Express Extract buffer and 2 μL KAPA Express Extract enzyme at 75 °C for 10 min, followed by 95 °C for 5 min to inactivate the protease. Lysates were briefly centrifuged, and 1–2 μL of the supernatant was used directly as the PCR template. PCR amplification was performed using KOD One PCR Master Mix (KMM-201, TOYOBO). Each 50 μL reaction contained 25 μL KOD One Master Mix, 21 μL nuclease-free water, 1.5 μL human PRDX4 forward primer (5′-GAAGAGGAGTGCCACTTCTACG-3′), 1.5 μL human PRDX4 reverse primer (5′-AAGAGACCTCTAAGAGTGTGGCC-3′), and 1 μL extracted DNA. Reaction mixtures were prepared on ice and briefly mixed before loading into PCR tubes. The PCR procedure is as follows: 98 °C, 5 min; 30 cycles of: 98 °C, 10 sec; 60 °C, 5 sec; 72 °C, 1 sec; 72 °C, 7 min; 12 °C, hold. PCR products were resolved on 1.5–2% agarose gels prepared with 1×TAE buffer. Agarose was dissolved by microwave heating, cooled to ∼60 °C, mixed with Midori Green Direct nucleic acid stain, and poured into molds. Electrophoresis was performed at 100 V for 30 min in 1×TAE buffer. DNA bands were visualized using a gel documentation system under blue light or UV illumination.

### Public database analysis

To evaluate the expression pattern and prognostic significance of PRDX4 at the mRNA level, publicly available datasets were analyzed. PRDX4 mRNA expression in pancreatic cancer and normal pancreatic tissues was assessed using TCGA-based data through online platforms. The association between PRDX4 expression and disease-free survival (DFS) was analyzed using the Kaplan-Meier Plotter database (https://kmplot.com/analysis/). Patients were stratified into high and low expression groups based on the auto-selected best cutoff provided by the database. Survival curves were generated using the Kaplan-Meier method, and statistical significance was determined by the log-rank test. Hazard ratios (HRs) with 95% confidence intervals (CIs) were calculated automatically by the database.

### Statistical analysis

The statistical analyses were performed using SPSS 26.0 (IBM Corp., Armonk, NY, USA) and GraphPad Prism 10.0 (GraphPad Software, San Diego, CA, USA). ROC curve analysis was used to determine the optimal cut-off value of PRDX4 expression based on the Youden index. To further assess the robustness of the ROC derived cut-off, a bootstrap resampling procedure with 2000 iterations was performed, in which the optimal cut-off value was recalculated in each resampled dataset. For clinicopathological data, categorical variables were compared using the Chi-square or Fisher’s exact test, and survival outcomes were analyzed by the Kaplan-Meier method with log-rank test. Prognostic factors were evaluated using Cox proportional hazards models, and results were expressed as HRs with 95% CIs. Variables included in multivariable Cox models were selected based on a combination of statistical significance in univariate analyses and established clinical relevance, while also considering the events-per-variable (EPV) principle to minimize overfitting. To assess the robustness of the prognostic effect of PRDX4 and to improve comparability between the 3- and 5-year DSS models, an additional sensitivity analysis was performed using a fixed multivariable Cox model with the same prespecified covariates for both endpoints, including age, gender, tumor differentiation, TNM stage, and PRDX4 expression. The proportional hazards assumption was assessed using Schoenfeld residuals. For in vitro assays (CCK-8, Transwell, ROS assay and Western blot quantification), data were obtained from at least three independent experiments and expressed as mean±standard deviation (SD). Group comparisons were performed using two-tailed Student’s *t*-test or one-way analysis of variance (ANOVA) as appropriate. For in vivo studies, tumor volumes, necrotic areas, and microvessel densities were analyzed using two-way ANOVA with Bonferroni post hoc correction. A two-tailed P < 0.05 was considered statistically significant.

## Results

### Expression patterns and clinicopathological associations of PRDX4

A total of 128 patients with histologically confirmed PDAC were included in the study. The baseline clinicopathological characteristics are summarized in Table 1. Most patients were older adults, with 110 individuals aged >60 years and 18 aged≤60 years. The gender distribution was nearly balanced (male: 66; female: 62). The majority of tumors were located in the pancreatic head (83 cases), while 45 arose in the body or tail. Tumor size exceeded 20 mm in 87 patients, whereas 41 had tumors≤20 mm. Histologically, 55 tumors were well-differentiated, 63 were moderately differentiated, and 10 showed poor differentiation. Lymphatic vessel invasion was present in 106 patients, vascular invasion in 111, and perineural invasion in 102. Regarding tumor staging, most cases were classified as pT3 or pT4 (82 and 30 cases, respectively), and 75 patients had nodal metastasis (pN1–2). Distant metastasis (pM1) was identified in 6 cases. According to the AJCC TNM classification, 15 patients were stage 0-I, 79 were stage II, 28 were stage III, and 6 were stage IV. Based on the ROC-derived cut-off, PRDX4 expression was categorized as low in 65 patients and high in 63. For survival analyses, 65 DSS events occurred within 3 years, and 80 DSS events were documented within 5 years of follow-up.

**Table 1:** Baseline clinicopathological characteristics of patients with PDAC who underwent surgical resection, including curative intent and palliative procedures (n = 128).

Variables	Patients (n = 128)
Age	
>60	110
≤60	18
Gender	
Female	62
Male	66
Location	
Head	83
Body/Tail	45
Tumor size (mm)	
>20	87
≤20	41
Differentiation	
Well	55
Moderately	63
Poorly	10
Lymphatic vessel invasion	
Present	106
Absent	22
Vascular invasion	
Present	111
Absent	17
Perineural invasion	
Present	102
Absent	26
pT	
Tis, T1	13
T2	3
T3	82
T4	30
pN	
N0	53
N1–2	75
pM	
M0	122
M1	6
TNM stage	
0∼Ⅰ	15
Ⅱ	79
Ⅲ	28
Ⅳ	6
PRDX4 expression	
Low	65
High	63
PDAC specific deaths during follow-up	
3-year DSS	65
5-year DSS	80

**Fig. 1:**
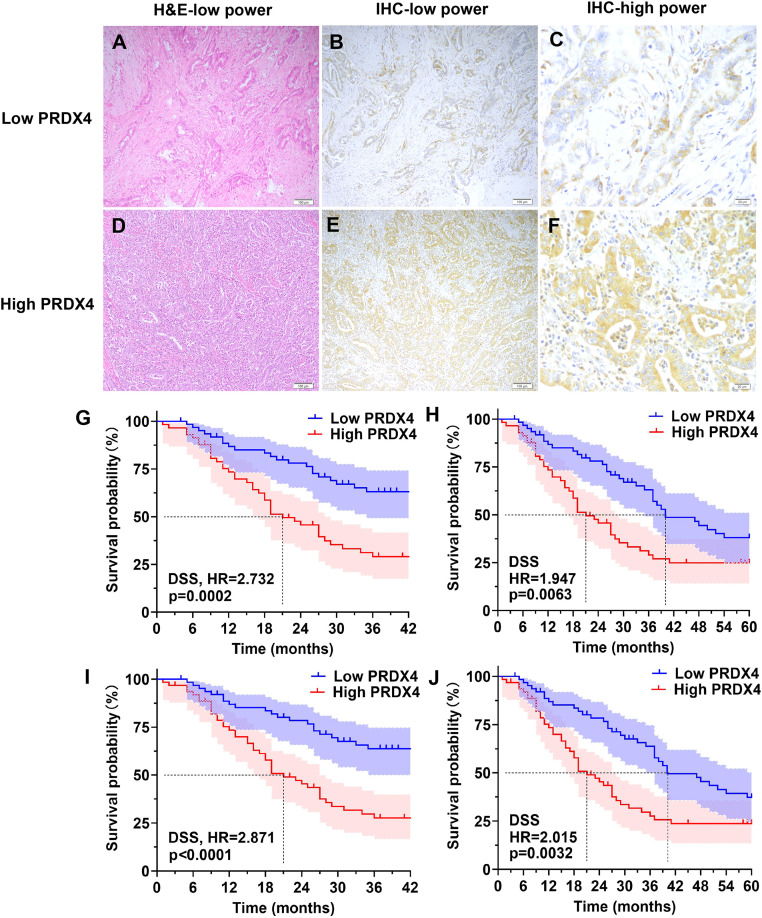
Histopathological features and prognostic significance of PRDX4 expression in PDAC. Representative hematoxylin and eosin (H&E) and IHC images showing differential PRDX4 expression in PDAC. (A-C) Tumors with low PRDX4 expression exhibit typical glandular architecture with moderate desmoplasia on H&E (A) and a small number of cytoplasmic PRDX4 staining cells under both low-power (B) and high-power (C) magnifications. (D-F) Tumors with high PRDX4 expression display more irregular glandular structures and invasive growth on H&E (D), accompanied by a large number of diffuse cytoplasmic PRDX4 immunoreactivity under low- (E) and high-power (F) fields. Scale bars: 100 μm (A, B, D, E) and 20 μm (C, F). (G-J) Kaplan-Meier analysis of DSS according to PRDX4 expression in PDAC. Patients were stratified into high and low PRDX4 expression groups based on immunohistochemical scoring. (G) Patients with high PRDX4 expression showed significantly poorer 3-year DSS compared with those with low PRDX4 expression in the excluding cohort (log-rank, *P* = 0.0002). (H) The association between high PRDX4 expression and reduced survival remained significant for 5-year DSS in the excluding cohort (log-rank, *P* = 0.0063). (I) High PRDX4 expression was still associated with significantly poorer 3-year DSS in the entire cohort (log-rank, *P* < 0.0001). (J) Consistently, the adverse prognostic impact of high PRDX4 expression persisted for 5-year DSS in the entire cohort (log-rank, *P* = 0.0032).

Representative images in Fig. 1 illustrate the histological and immunohistochemical features of PRDX4 in pancreatic cancer tissues. Hematoxylin and eosin (H&E) sections demonstrated the typical glandular architecture and cytological heterogeneity of pancreatic cancer (Fig. 1A, D). In tumors with low PRDX4 expression, immunostaining revealed a small number of cytoplasmic signals under both low- and high-power magnifications (Fig. 1B, C). In contrast, high-expression tumors exhibited a large number of diffuse cytoplasmic staining across the tumor nests (Fig. 1E, F), consistent with enhanced PRDX4 immunoreactivity in more aggressive lesions.

The associations between PRDX4 expression and various clinicopathological parameters are summarized in Table 2. Notably, high PRDX4 expression was not only significantly correlated with advanced pT stage (*P* < 0.001), lymph node metastasis (*P* < 0.001), and higher TNM stage (*P* < 0.001), but also showed a striking association with aggressive invasive features. Specifically, 93.7% (59/63) of patients in the high PRDX4 group exhibited lymphatic vessel invasion, and 100% (63/63) demonstrated vascular invasion, both of which were significantly higher than those in the low PRDX4 group (*P* = 0.001 and *P* < 0.001, respectively). These findings are consistent with the aggressive phenotypes observed in subsequent in vitro and in vivo analyses.

**Table 2:** Correlations Between PRDX4 Expression and Clinicopathological Parameters in PDAC.

Variables	Low PRDX4 (n = 65)	High PRDX4 (n = 63)	p Value
**Age**			
>60	55	55	0.662
≤60	10	8	
**Gender**			
Female	33	29	0.592
Male	32	34	
**Location**			0.426
Head	40	43	
Body/Tail	25	20	
**Tumor size (mm)**			
>20	41	46	0.228
≤20	24	17	
**Differentiation**			
Well	28	27	0.371
Moderately	34	29	
Poorly	3	7	
**Lymphatic vessel invasion**			
Present	47	59	0.001
Absent	18	4	
**Vascular invasion**			
Present	48	63	<0.001
Absent	17	0	
**Perineural invasion**			
Present	46	56	0.011
Absent	19	7	
**pT**			
Tis, T1-T2	15	1	<0.001
T3-T4	50	62	
**pN**			
N0	37	16	<0.001
N1–2	28	47	
**pM**			
M0	64	58	0.087
M1	1	5	
**TNM stage**			
0∼ⅡA	33	11	<0.001
ⅡB-Ⅳ	32	52	

### Correlation of PRDX4 with NRF2 and HO-1 expression in clinical PDAC specimens

We investigated the relationship between PRDX4 and redox-related downstream targets in clinical PDAC tissues, immunohistochemical staining for NRF2 and HO-1 was performed. Representative images demonstrated that tumors with high PRDX4 expression exhibited correspondingly increased NRF2 and HO-1 positive staining cells, whereas tumors with low PRDX4 expression showed relatively decreased positive staining (fig. S2A). Spearman correlation analysis revealed a significant positive correlation between PRDX4 and NRF2 expression (r = 0.6003, *P* < 0.001), as well as between PRDX4 and HO-1 expression (r = 0.7887, *P* < 0.001) (fig. S2B-C).

### Prognostic significance of PRDX4 expression in PDAC

Public database analysis revealed that PRDX4 mRNA expression was significantly lower in pancreatic tumor tissues compared with normal pancreatic tissues. To evaluate the prognostic significance of PRDX4 expression in a clinically relevant setting, survival analyses were primarily performed after excluding patients with distant metastasis (pM1). In this restricted cohort, univariate Cox regression analysis identified tumor location, tumor size, tumor differentiation, vascular invasion, TNM stage and PRDX4 expression as factors associated with 3-year DSS. In multivariate analysis adjusting for established clinicopathological variables, tumor differentiation (HR = 1.781, *P* = 0.005) and PRDX4 expression remained independently associated with poorer 3-year DSS (HR = 1.725, *P* = 0.045) (Table 3). Similarly, for 5-year DSS, PRDX4 expression was significantly associated with reduced survival in both univariate and multivariate analyses (multivariate HR = 1.578, *P* = 0.031), along with tumor differentiation and TNM stage (Table 3). The proportional hazards assumption was satisfied for all multivariable Cox models, and no significant multicollinearity was detected among the included covariates, supporting the robustness of the model estimates (Table S3). Kaplan-Meier survival analysis after exclusion of pM1 patients further demonstrated that patients with high PRDX4 expression exhibited significantly poorer 3- and 5-year DSS compared with those with low expression in the pM1-excluded cohort (log-rank *P* = 0.0002, 0.0063, respectively) (Fig. 1G, H).

**Table 3:** Univariate and Multivariate Cox Regression Analyses of 3- and 5-Year DSS According to Clinicopathological Factors after excluding pM1 patients.

3-years DSS						
Variables	Univariate			Multivariate		
	Hazard ratio	95% CI	p Value	Hazard ratio	95% CI	p Value
Age	1.594	0.856–2.379	0.225			
Gender	1.585	0.959–2.620	0.073			
Location	0.550	0.314–0.962	0.036	0.592	0.334–1.050	0.073
Tumor size	1.885	1.038–3.423	0.037	1.525	0.817–2.849	0.185
Differentiation	1.699	1.143–2.527	0.009	1.781	1.194–2.657	0.005
Lymphatic vessel invasion	1.518	0.836–3.137	0.101			
Vascular invasion	1.275	1.086–3.877	0.029	1.477	0.630–3.745	0.263
Perineural invasion	0.618	0.305–1.253	0.182			
TNM stage	2.885	1.534–5.424	0.001	1.702	0.824–3.516	0.151
PRDX4 expression	2.448	1.458–4.111	0.001	1.725	1.015–2.987	0.045
**5-years DSS**	Hazard ratio	95% CI	p Value	Hazard ratio	95% CI	p Value
Age	1.502	0.330–2.094	0.083			
Gender	1.508	0.960–2.371	0.075			
Location	0.593	0.363–0.969	0.037	0.620	0.376–1.023	0.061
Tumor size	1.519	0.917–2.516	0.104			
Differentiation	1.465	1.016–2.112	0.041	1.685	1.157–2.453	0.006
Lymphatic vessel invasion	1.457	1.219–2.951	0.036	1.673	0.648–3.830	0.438
Vascular invasion	1.348	1.040–2.863	0.023	1.546	0.650–2.987	0.359
Perineural invasion	0.559	0.287–1.087	0.087			
TNM stage	2.063	1.236–3.445	0.006	1.460	0.828–2.577	0.191
PRDX4 expression	1.791	1.137–2.822	0.012	1.578	1.023–3.076	0.031

To further assess the robustness of these findings, we performed additional survival analyses on all cases, including pM1 patients. In this entire cohort, univariate and multivariate Cox regression analyses yielded results consistent with those obtained in the excluded cohort. High PRDX4 expression remained significantly associated with poorer 3-year DSS in both univariate (HR = 2.585, *P* *<* 0.001) and multivariate analyses (HR = 1.820, *P* = 0.030), after adjustment for established clinicopathological factors (Table 4). Similarly, for 5-year DSS, PRDX4 expression continued to emerge as an independent adverse prognostic factor in multivariate analysis (HR = 1.600, *P* = 0.047) (Table 4). The proportional hazards assumption was satisfied for all multivariable Cox models, and no significant multicollinearity was detected among the included covariates, supporting the robustness of the model estimates (Table S4). Kaplan-Meier survival analyses further confirmed these results. Patients with high PRDX4 expression exhibited significantly worse 3-year and 5-year DSS compared with those with low expression (log-rank *P* < 0.01 for both), as shown in Fig. 1I and J. In an additional sensitivity analysis using a fixed multivariable Cox model with the same prespecified covariates for both 3-year and 5-year DSS, the prognostic effect of PRDX4 remained materially unchanged (Table S5). We also analyzed the association between PRDX4 expression levels and prognosis using TCGA-based data (Kaplan-Meier Plotter). The results showed that patients with high PRDX4 expression had significantly worse disease-free survival (DFS) compared with those with low PRDX4 expression (fig. S3, *P* < 0.05).

**Table 4:** Univariate and Multivariate Cox Regression Analyses of 3- and 5-Year DSS According to Clinicopathological Factors.

3-years DSS						
Variables	Univariate			Multivariate		
	Hazard ratio	95% CI	p Value	Hazard ratio	95% CI	p Value
Age	1.317	0.832–2.229	0.139			
Gender	1.692	0.932–2.773	0.065			
Location	0.580	0.336–1.035	0.062			
Tumor size	2.045	1.131–3.699	0.018	1.513	0.822–2.785	0.184
Differentiation	1.677	1.133–2.480	0.010	1.785	1.205–2.644	0.004
Lymphatic vessel invasion	1.526	0.847–3.266	0.061			
Vascular invasion	2.155	1.077–3.588	0.018	1.842	0.625–3.569	0.207
Perineural invasion	0.566	0.280–1.145	0.113			
TNM stage	2.908	1.552–5.449	0.001	1.871	1.021–3.692	0.039
PRDX4 expression	2.585	1.548–4.315	<0.001	1.820	1.060–3.124	0.030
**5-years DSS**	Hazard ratio	95% CI	p Value	Hazard ratio	95% CI	p Value
Age	1.509	0.244–2.060	0.071			
Gender	1.569	0.908–2.442	0.066			
Location	0.608	0.376–1.183	0.125			
Tumor size	1.577	0.964–2.581	0.070			
Differentiation	1.462	1.020–2.096	0.038	1.528	1.067–2.188	0.021
Lymphatic vessel invasion	1.325	0.231–2.929	0.322			
Vascular invasion	1.983	0.898–2.856	0.102			
Perineural invasion	0.559	0.296–1.057	0.074			
TNM stage	2.101	1.264–3.493	0.004	1.890	1.113–3.210	0.019
PRDX4 expression	1.851	1.187–2.888	0.007	1.600	1.006–2.544	0.047

### Functional effects of PRDX4 overexpression on PDAC cell proliferation and migration

**Fig. 2:**
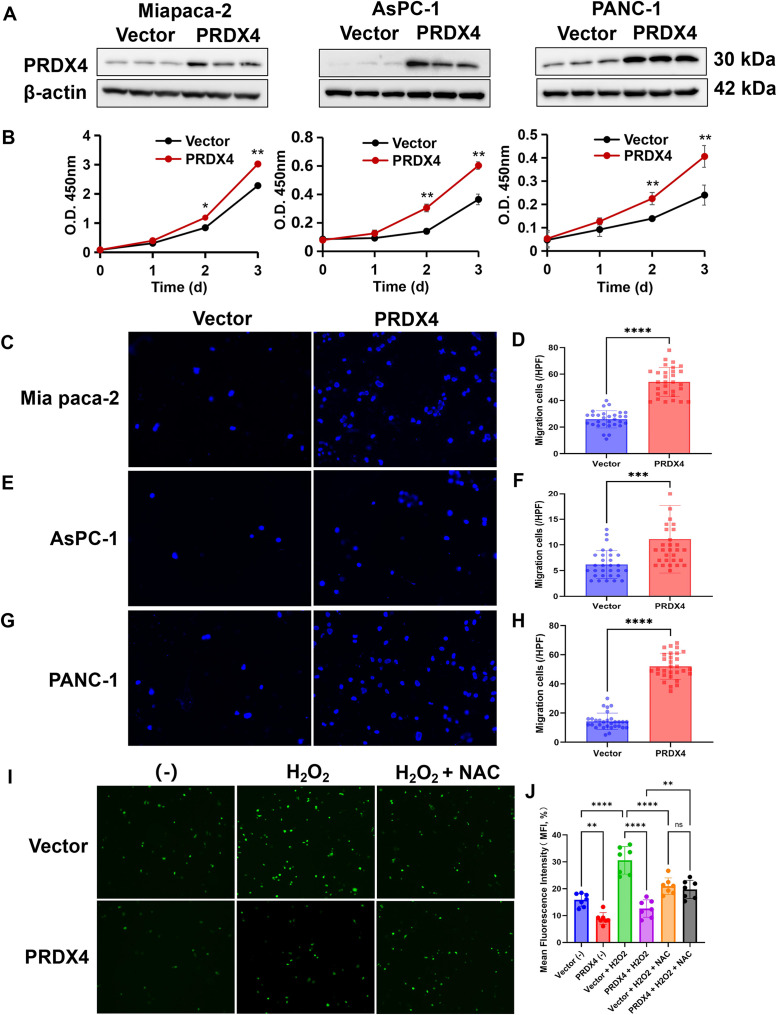
PRDX4 overexpression promotes PDAC cell proliferation and migration and modulates intracellular ROS levels. (A) Analyses of the PRDX4 expression after transfected pCMV and human PRDX4 plasmid DNA into Mia paca-2, AsPC-1, PANC-1 cell lines were confirmed by Western blot, PRDX4 overexpression in MiaPaCa-2, AsPC-1, and PANC-1 cells following transfection with PRDX4 expression plasmid. (B) Cell proliferation was assessed using the CCK-8 assay at indicated time points after transfection. Data are presented as mean ± SD from three independent experiments (*P < 0.05, ^⁎⁎^*P* < 0.01). (C, E, G) Representative images of migrated cells in transwell migration assays in MiaPaCa-2, AsPC-1, and PANC-1 cells following PRDX4 overexpression. (D, F, H) Quantitative analysis of migrated cells per high-power field. Data are presented as mean±SD from three independent experiments (^⁎⁎⁎^*P* < 0.001, ^⁎⁎⁎⁎^*P* < 0.0001). (I) Representative fluorescence images of intracellular ROS levels detected using the DCFH-DA probe in vector control and PRDX4-overexpressing PANC-1 cells under basal conditions (-), after H_2_O_2_ treatment, and after combined H_2_O_2_ and NAC treatment. Cells were treated with H_2_O_2_ (200μM, 30 min) to induce oxidative stress. For antioxidant rescue experiments, cells were pretreated with NAC for 1 h prior to H_2_O_2_ exposure. Scale bars, 200μm. (J) Quantitative analysis of intracellular ROS levels expressed as MFI. Under basal conditions, PRDX4-overexpressing cells exhibited significantly lower ROS levels compared with vector controls. H_2_O_2_ treatment significantly increased ROS levels in both groups; however, PRDX4-overexpressing cells showed significantly attenuated ROS accumulation compared with vector control cells. NAC treatment reduced ROS levels under oxidative stress conditions and largely diminished the difference between vector and PRDX4-overexpressing cells. Data are presented as mean±SD from at least three independent experiments. Statistical significance was determined using Student’s *t*-test or one-way ANOVA as appropriate (*P < 0.05, ^⁎⁎^*P* < 0.01, ^⁎⁎⁎⁎^*P* < 0.0001; ^ns^*P* > 0.05).

Baseline PRDX4 protein expression was detectable in all three cell lines, without extreme inter-cell-line variability. To determine the functional role of PRDX4 in PDAC, we examined the effects of its overexpression on cell proliferation and migration in three human PDAC cell lines (MiaPaCa-2, AsPC-1, and PANC-1). Western blot analysis confirmed successful PRDX4 overexpression following plasmid transfection compared with vector controls, as evidenced by markedly increased PRDX4 protein levels across all cell lines (Fig. 2A). Cell viability assessed by the CCK-8 assay demonstrated that PRDX4 overexpression significantly enhanced cell proliferation in a time dependent manner. In MiaPaCa-2 cells, optical density (OD) values at 450 nm increased more rapidly in the PRDX4 group than in vector controls, reaching approximately 3.0 by 72 h compared with ∼2.0 in the control group (*P* < 0.01) (Fig. 2B, left panel). Similar proliferative advantages were observed in AsPC-1 and PANC-1 cells, with statistically significant increases from 48 h onward (*P* < 0.01) (Fig. 2B, middle panel and right panel). To facilitate cross-cell-line comparison, proliferation data were additionally normalized to baseline (day 0) and presented as fold-change (fig. S4). This analysis demonstrated consistent proliferative trends across all three PDAC cell lines, with PRDX4 overexpression leading to a greater relative increase in cell proliferation compared with vector controls. Notably, while absolute OD values differed among cell lines, fold-change normalization confirmed that the pro-proliferative effect of PRDX4 was consistently observed in MiaPaCa-2, AsPC-1, and PANC-1 cells. To further investigate whether PRDX4 also affects the migratory potential of PDAC cells, transwell migration assays were performed under serum gradient conditions. In all three cell lines, PRDX4 overexpression markedly increased the number of migrated cells compared with vector-transfected controls (Fig. 2C, E, G). Quantitative analysis revealed that MiaPaCa-2 cells in the PRDX4 group exhibited nearly twice as many migrated cells per high-power field (mean≈60 vs. 35; ^⁎⁎⁎⁎^*P* < 0.0001) (Fig. 2D). Similar trends were observed in AsPC-1 (mean≈13 vs. 8; ^⁎⁎⁎^*P* < 0.001, Fig. 2F) and PANC-1 (mean≈65 vs. 35; ^⁎⁎⁎⁎^*P* < 0.0001, Fig. 2H) cells. Collectively, these results demonstrate that PRDX4 overexpression markedly enhances both the proliferative and migratory capacities of pancreatic cancer cells, supporting an association between PRDX4 overexpression and aggressive tumor-associated phenotypes in vitro.

### PRDX4 expression modulates intracellular ROS levels and redox responsiveness in PDAC cells

Intracellular ROS levels were evaluated using a fluorescence-based ROS detection assay to determine whether PRDX4 expression is associated with intracellular redox status. Under basal conditions, PRDX4-overexpressing PANC-1 cells exhibited consistently lower intracellular ROS levels compared with vector control cells, indicating enhanced intrinsic antioxidant capacity associated with PRDX4 expression (Fig. 2I). This observation was supported by quantitative analysis of mean fluorescence intensity (MFI), which demonstrated a significant reduction in ROS signal in PRDX4-overexpressing cells relative to controls (Fig. 2J). To further evaluate redox responsiveness under oxidative stress, cells were exposed to exogenous H₂O₂. H₂O₂ treatment markedly increased intracellular ROS levels in both vector control and PRDX4-overexpressing cells (Fig. 2I). However, quantitative MFI analysis revealed that PRDX4-overexpressing cells exhibited significantly attenuated ROS accumulation compared with vector control cells following H₂O₂ exposure, suggesting enhanced redox buffering capacity (Fig. 2J). To further determine whether PRDX4-associated redox alterations are linked to ROS scavenging, cells were treated with the antioxidant NAC. NAC treatment reduced intracellular ROS levels under oxidative stress conditions in both groups (Fig. 2I). Notably, NAC treatment markedly diminished the difference in ROS levels between PRDX4-overexpressing and vector control cells, as confirmed by MFI quantification, supporting the notion that PRDX4-mediated redox regulation is closely associated with intracellular ROS modulation (Fig. 2J).

To further validate the reproducibility of PRDX4-associated redox modulation across different PDAC cellular contexts, additional ROS assays were performed in MiaPaCa-2 cells. Consistent with the findings observed in PANC-1 cells, PRDX4 overexpression in MiaPaCa-2 cells was associated with reduced intracellular ROS levels under basal conditions. Following H_2_O_2_-induced oxidative stress, PRDX4-overexpressing cells exhibited attenuated ROS accumulation compared with vector control cells. Moreover, NAC treatment largely diminished the differences between PRDX4-overexpressing and control cells. Quantitative analysis of MFI confirmed these trends. These results further support that PRDX4-associated redox modulation is reproducible across multiple PDAC cell lines (fig. S5).

### PRDX4 overexpression selectively modulates MAPK and PI3K/AKT signaling and promotes EMT-associated phenotypes in PDAC cells

**Fig. 3:**
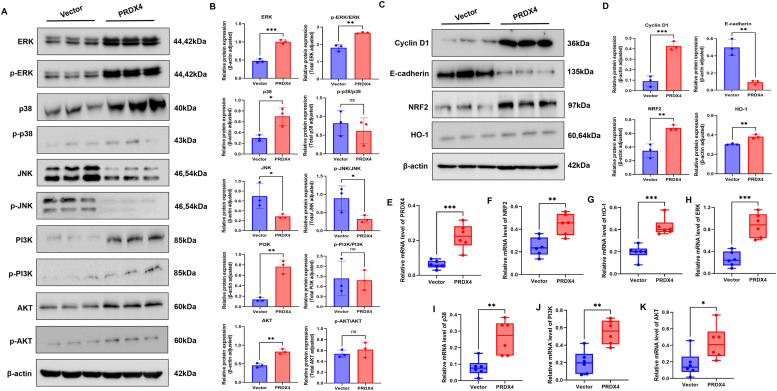
PRDX4 overexpression is associated with alterations in signaling-, cell cycle-, and redox-related protein and mRNA expression in PDAC cells. (A) Representative Western blot images showing protein expression levels of ERK, p-ERK, p38, p-p38, JNK, p-JNK, PI3K, p-PI3K, AKT, and p-AKT in vector control and PRDX4-overexpressing cells. (B) Quantification of MAPK and PI3K/AKT pathway-related proteins. PRDX4 overexpression was associated with increased ERK phosphorylation, reduced JNK phosphorylation, and increased total protein levels of p38, PI3K, and AKT, while the corresponding phosphorylation levels of p38, PI3K, and AKT did not show statistically significant differences. (C) Representative Western blot images of Cyclin D1, E-cadherin, NRF2, and HO-1 expression in vector and PRDX4-overexpressing cells. (D) Quantification of Cyclin D1, E-cadherin, NRF2, and HO-1 protein expression. (E-K) RT-qPCR analysis showing relative mRNA expression levels of PRDX4 (E), NRF2 (F), HO-1 (G), ERK (H), p38 (I), PI3K (J), and AKT (K) in vector control and PRDX4-overexpressing cells. PRDX4 overexpression was associated with increased mRNA expression levels of these genes. Statistical analysis was performed using unpaired *t*-test (*P < 0.05, ^⁎⁎^*P* < 0.01, ^⁎⁎⁎^*P* < 0.001, ^ns^*P* > 0.05).

To further elucidate the molecular mechanisms underlying the consistent pro-proliferative and pro-migratory effects of PRDX4 observed across multiple PDAC cell lines, we next investigated PRDX4-associated signaling alterations in PANC-1 cells as a representative model. As mentioned above, Western blot analysis confirmed successful PRDX4 upregulation compared with vector controls. PRDX4 overexpression led to a distinct changes of intracellular signaling networks. Specifically, ERK/p-ERK, p38, PI3K, and AKT exhibited elevated levels, whereas JNK/p-JNK expression was markedly suppressed, indicating a context dependent re-balancing within the MAPK cascade (Fig. 3A, B). Consistent with the enhanced migratory phenotype, PRDX4 overexpression resulted in a pronounced downregulation of the epithelial marker E-cadherin, suggesting the induction of EMT-associated cellular plasticity. In parallel, expression of the cell cycle regulator Cyclin D1 was significantly increased, supporting a pro-proliferative role of PRDX4 in PDAC cells. Moreover, PRDX4 overexpression was accompanied by upregulation of NRF2 and its downstream antioxidant enzyme HO-1, suggesting association with an antioxidant stress-adaptive program (Fig. 3C, D). To assess whether PRDX4-associated signaling changes observed in PANC-1 cells were reproducible in other PDAC models, selected key proteins were further examined in MiaPaCa-2 cells. As shown in fig. S6, PRDX4 overexpression in MiaPaCa-2 cells led to increased ERK phosphorylation, elevated AKT, elevated p38 and NRF2 accumulation, accompanied by downregulation of E-cadherin and upregulation of HO-1. Although the magnitude of signaling alterations varied between cell lines, the overall trends were consistent with those observed in PANC-1 cells, supporting a conserved role of PRDX4 in modulating MAPK-associated signaling, epithelial plasticity, and redox-adaptive responses across heterogeneous PDAC cellular contexts.

### PRDX4 overexpression is associated with transcriptional alterations in NRF2, HO-1 and MAPK/PI3K-AKT-related genes

To further determine whether PRDX4 overexpression regulates gene expression at the transcriptional level, RT-qPCR analysis was performed in PANC-1 cells. As shown in Fig. 3E, PRDX4 mRNA expression was significantly increased in PRDX4-overexpressing cells compared with vector control cells (*P* < 0.05), confirming successful transcriptional upregulation. In addition, PRDX4 overexpression was associated with significantly increased mRNA expression levels of NRF2, HO-1, ERK, p38, PI3K, and AKT compared with vector control cells (Fig. 3F-K, *P* < 0.05).

### In vivo effects of PRDX4 overexpression on pancreatic tumor growth and response to GEM

**Fig. 4:**
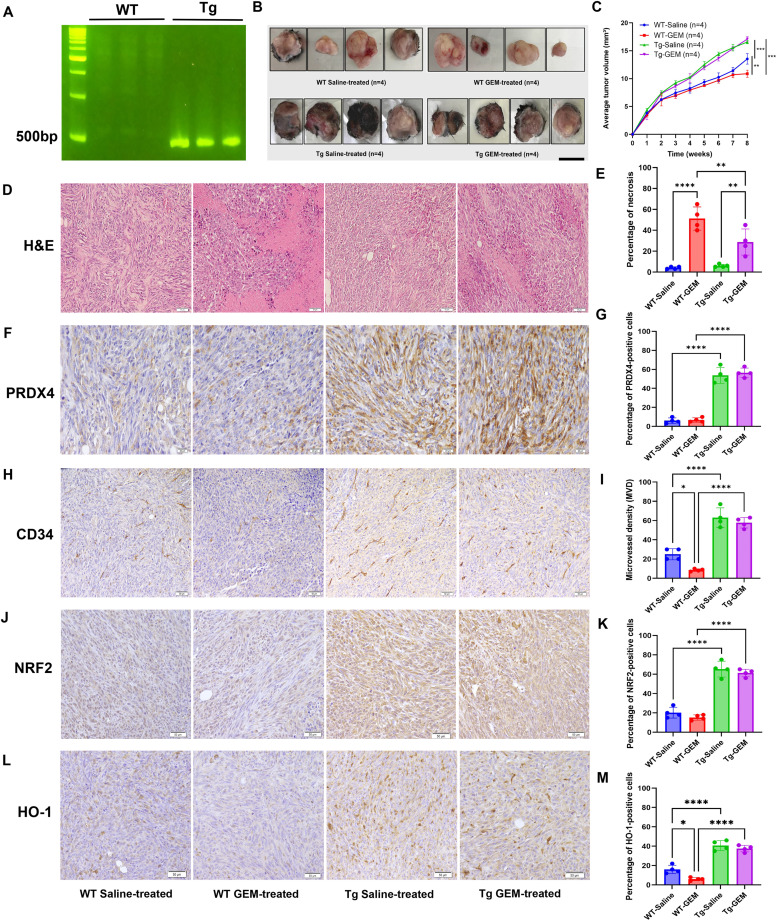
PRDX4 overexpression confers GEM resistance and sustains angiogenesis in vivo. (A) Genotyping PCR confirming successful establishment of Tg mice without genotype abnormalities. (B) Representative images of syngeneic subcutaneous tumor excised from WT and Tg mice after 4 weeks of treatment with either saline or GEM (50 mg/kg/week, intraperitoneally; scale bar: 2 cm). (C) Tumor growth curves showing that GEM markedly inhibited tumor growth in WT mice, whereas Tg mice exhibited attenuated responses and continued tumor enlargement. Data are presented as mean ± SD (^⁎⁎^*P* < 0.01, ^⁎⁎⁎^*P* < 0.001). (D) Representative H&E stained tumor sections (Scale bars = 50 μm). GEM-treated WT tumors exhibited extensive necrosis, while tumors developed in Tg mice displayed limited necrotic areas and predominant viable tumor regions, consistent with reduced responsiveness to GEM. (E) Quantitative analysis of necrotic area percentage demonstrating significantly lower necrosis in Tg groups compared with WT groups (^⁎⁎^*P* < 0.01, ^⁎⁎⁎⁎^*P* < 0.0001). (F) Immunohistochemical staining of PRDX4 showing strong cytoplasmic immunoreactivity in tumors developed in Tg mice compared with WT mice (Scale bars = 20 μm). (G) Quantification of PRDX4-positive cells confirming markedly higher expression levels in Tg groups (^⁎⁎⁎⁎^*P* < 0.0001). (H) CD34 immunostaining illustrating microvessel density (MVD) (Scale bars = 50 μm). WT tumors exhibited sparse vasculature, which decreased further after GEM treatment, while tumors developed in Tg mice maintained abundant neovessels irrespective of treatment. (I) Quantitative analysis of MVD showing significantly higher vascular density in Tg group than WT group, with GEM induced suppression observed only in WT tumors (*P < 0.05, ^⁎⁎⁎⁎^*P* < 0.0001). (J) Immunohistochemical staining of NRF2 showing strong nuclear and cytoplasmic immunoreactivity in tumors developed in Tg mice compared with WT mice (Scale bars = 20 μm). (K) Quantification of NRF2-positive cells confirming markedly higher expression levels in Tg groups (^⁎⁎⁎⁎^*P* < 0.0001). (L) Immunohistochemical staining of HO-1 showing strong cytoplasmic immunoreactivity in tumors developed in Tg mice compared with WT mice (Scale bars = 20 μm). (M) Quantification of HO-1-positive cells confirming markedly higher expression levels in Tg groups (*P < 0.05, ^⁎⁎⁎⁎^*P* < 0.0001).

To further validate the oncogenic role of PRDX4 in vivo, we established syngeneic subcutaneous tumor model using Pan02 cells in C57BL/6 mice, including both WT and Tg mice. As shown in Fig. 4A, genotyping polymerase chain reaction (PCR) confirmed the successful establishment of Tg mice without any genotype abnormalities. All mice successfully developed tumors. During a 4-week period, GEM was administered once tumors reached 150–200 mm³. Importantly, tumor volumes were comparable between WT and Tg mice at the time of GEM initiation (*P* > 0.05), indicating that subsequent differences in tumor growth reflected treatment response rather than baseline tumor burden. In WT mice, GEM treatment significantly inhibited tumor growth compared with saline controls, with average tumor volume markedly reduced after 4 weeks (*P* < 0.05). By contrast, Tg mice showed attenuated responses to GEM, as tumor volumes continued to increase despite treatment, and statistical differences between GEM and saline treated Tg groups were not significant (Fig. 4B, C). Comparisons across genotypes revealed that Tg mice exhibited larger tumor burdens and greater resistance to GEM relative to WT counterparts (*P* < 0.05). Histopathological evaluation demonstrated that GEM treated WT tumors displayed more extensive necrosis compared to saline controls, consistent with enhanced chemosensitivity. In Tg mice, however, GEM treated tumors showed limited necrosis, and viable tumor regions remained predominant, supporting the observation of therapeutic resistance (Fig. 4D, E). We also performed immunohistochemical assessments and found that tumors grown in Tg mice exhibited significantly higher PRDX4 levels compared to those in WT mice. This elevated PRDX4 immunoreactivity may result from the recruitment of PRDX4 high stromal cells or the induction of PRDX4 expression in tumor cells by the Tg mice microenvironment. However, because PRDX4 is constitutively overexpressed in host tissues of Tg mice and immunohistochemistry does not allow definitive discrimination between tumor cell-derived and host stromal PRDX4, the observed increase likely reflects a combined contribution from both compartments. Importantly, GEM treatment did not significantly alter PRDX4 expression levels within either the WT or Tg groups (Fig. 4F, G). In addition, tumor angiogenesis was evaluated by CD34 immunostaining. WT tumors treated with saline exhibited relatively sparse microvessels, and GEM administration further reduced vascular density, in line with inhibited tumor growth. In contrast, tumors developed in Tg mice showed markedly increased CD34 positive neovessels under saline conditions, indicative of enhanced angiogenic activity driven by PRDX4 overexpression. Notably, despite GEM treatment, tumors developed in Tg mice retained abundant and dense vascular networks (Fig. 4H), suggesting that angiogenesis persisted and may contribute to the reduced chemosensitivity observed in these mice. Quantitative analysis confirmed significantly higher microvessel density in Tg group compared with WT group, while GEM related vascular suppression occurred only in WT tumors (Fig. 4I). NRF2 immunohistochemical staining showed relatively high expression in the WT-Saline group and a slight decrease in the WT-GEM group. In the Tg-Saline group, NRF2 expression was higher than that in WT-Saline; And in the Tg-GEM group, NRF2 expression was higher than that in WT-GEM. The Tg-GEM group showed NRF2 expression levels comparable to or slightly lower than those in the Tg-Saline group (Fig. 4J-K). Similarly, HO-1 staining demonstrated high expression in the WT-Saline group and decrease in the WT-GEM group. The Tg-Saline group exhibited higher HO-1 expression compared with WT-Saline, while the Tg-GEM group showed HO-1 levels that remained higher than those in WT-GEM (Fig. 4L-M).

**Fig. 5:**
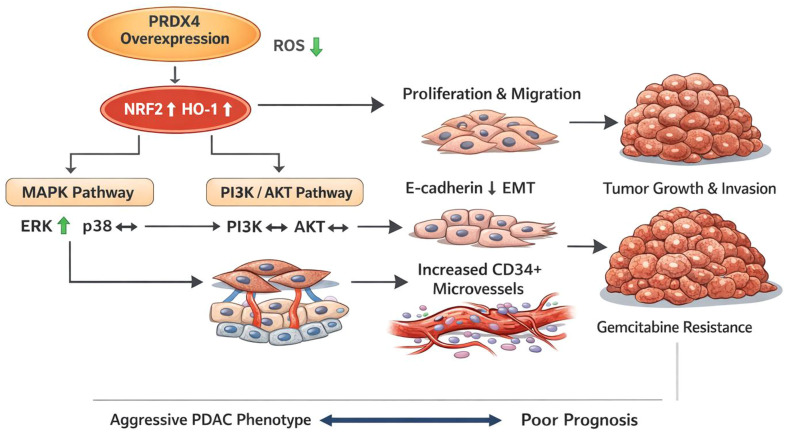
Proposed working model illustrating PRDX4-associated redox adaptations and tumor-related phenotypic changes in PDAC. Based on integrated in vitro and in vivo observations, PRDX4 overexpression is associated with altered intracellular redox status and changes in the expression of signaling- and antioxidant-related proteins, including ERK, NRF2, and HO-1. These molecular alterations coincide with increased tumor cell proliferation and migration in vitro, enhanced tumor growth and reduced responsiveness to GEM in vivo, and vascular-related features observed in tumor tissues. This schematic summarizes the associations identified in the present study and does not distinguish tumor cell–intrinsic from microenvironment-mediated effects.

Based on the integrated in vitro and in vivo findings, we propose a working model in which PRDX4 overexpression is associated with coordinated redox-related adaptations and tumor-associated phenotypic changes, including altered oncogenic signaling related protein expression, vascular-related features, and reduced responsiveness to GEM in PDAC (Fig. 5).

## Discussion

This study provides integrated clinical and experimental evidence positioning PRDX4 as a redox-associated regulator linking intracellular oxidative stress adaptation, supported by ROS measurement and antioxidant rescue experiments, to oncogenic signaling, tumor progression, and chemoresistance in PDAC. High PRDX4 expression was strongly associated with aggressive clinicopathological features and independently associated with poor DSS. Functionally, PRDX4 overexpression promoted PDAC cell proliferation and migration and was associated with selective shifts in MAPK- and PI3K/AKT-related signaling. In vivo, PRDX4 overexpression coincided with sustained tumor angiogenesis, reduced tumor necrosis, and attenuated responsiveness to GEM, consistent with previous evidence linking PRDX4 to vascular-associated tissue remodeling [29]. Together, these findings suggest that PRDX4 functions not only as an antioxidant enzyme but also as a redox-associated signaling modulator in PDAC.

As summarized in Fig. 5, our data support a model in which PRDX4 overexpression is associated with coordinated redox-related adaptations and tumor associated phenotypic changes. Oxidative stress is a fundamental feature of PDAC driven by oncogenic KRAS signaling, metabolic reprogramming, hypoxia, and TME interactions [30]. Although ROS can induce cytotoxicity, cancer cells often maintain ROS at sublethal levels through tightly controlled redox buffering systems to sustain proliferation and survival [31]. Within the ER lumen, PRDX4 consumes H_2_O_2_ generated during oxidative protein folding and contributes to disulfide bond formation via interactions with PDI, thereby maintaining ER redox balance and proteostasis [32]. This physiological role provides a framework for interpreting our findings that PRDX4 overexpression is associated with altered intracellular ROS levels, supported by NAC rescue experiments, increased NRF2 and HO-1 expression, and coordinated changes in oncogenic signaling-related protein expression. Rather than establishing direct mechanistic causality, these findings, together with antioxidant rescue-based validation of ROS modulation, support an association between elevated PRDX4 expression and altered redox-adaptive features in PDAC cells, coinciding with increased tumor cell proliferation, migration, tumor growth in vivo, and reduced responsiveness to GEM. Although direct mechanistic links were not established, these observations remain biologically and clinically meaningful in PDAC progression and therapeutic resistance.

PRDX4 overexpression was also associated with differential modulation of MAPK- and PI3K/AKT-related proteins. Increased ERK phosphorylation and reduced JNK phosphorylation were consistently observed, whereas p38, PI3K, and AKT primarily showed total protein level and mRNA level changes without clear phosphorylation differences. This pattern is consistent with a PRDX4-associated shift within the MAPK network potentially influenced by intracellular redox balance [33] and aligns with reports indicating that ROS scavenging favors ERK and AKT signaling while suppressing JNK activation [34]. Our data do not support direct enzymatic interaction between PRDX4 and these kinases. Instead, PRDX4-associated modulation of intracellular redox tone may influence upstream kinase activity and redox-sensitive phosphatases. Consistent with these signaling changes, PRDX4 overexpression was accompanied by increased Cyclin D1 and reduced E-cadherin expression, supporting enhanced cell cycle progression and epithelial plasticity. Functional assays across multiple PDAC cell lines demonstrated consistent proliferative and migratory effects. In addition, PRDX4 overexpression was associated with increased NRF2 and HO-1 protein and mRNA expression. NRF2 is a key regulator of antioxidant and cytoprotective responses and has been implicated in PDAC progression and therapeutic resistance [35]. Although upstream regulatory mechanisms were not examined in the present study, NRF2 activity is known to be modulated by redox-sensitive post-translational modifications and MAPK-associated signaling pathways [36]. Notably, NRF2 protein appeared as multiple bands in our Western blot analyses. This observation is consistent with the complex post-translational regulation of NRF2, including phosphorylation, KEAP1-mediated ubiquitination, and proteasomal processing [36]. Under conditions of oxidative stress, NRF2 can escape degradation and accumulate in multiple molecular forms, which may manifest as distinct bands. In addition, partial degradation products may also contribute to this pattern. Together, these findings support a link between PRDX4 expression and enhanced antioxidant adaptive features in PDAC cells. Future studies incorporating ER stress markers (e.g., BiP/GRP78, CHOP, ATF4) and functional assays will be required to further elucidate whether PRDX4-associated signaling alterations are mediated through ER stress or UPR activation.

Although complementary loss-of-function approaches would further strengthen the mechanistic interpretation of this study, PRDX4 depletion experiments in PDAC may present unique biological and technical challenges. PRDX4 is an endoplasmic reticulum-associated antioxidant enzyme involved in oxidative protein folding, intracellular redox homeostasis, and ER proteostasis [12]. Therefore, abrupt or sustained PRDX4 depletion may induce broad oxidative stress, compensatory antioxidant responses, proteostatic disturbance, and impaired cell viability, potentially complicating interpretation of downstream ROS-associated and signaling-related changes. In addition, the discrepancy between relatively low PRDX4 mRNA expression in public transcriptomic datasets and high PRDX4 protein expression in a substantial subset of clinical PDAC specimens suggests that PRDX4 may be subject to post-transcriptional regulation or protein-stability-associated regulation in PDAC. These findings highlight the biological complexity of PRDX4 regulation and support the need for carefully designed future studies using transient siRNA-mediated knockdown, inducible depletion systems, rescue experiments, and time-course analyses.

In vivo, PRDX4 overexpression was associated with accelerated tumor growth and reduced responsiveness to GEM. Tumors in PRDX4 Tg mice exhibited increased growth, reduced necrosis, and attenuated GEM sensitivity compared with WT hosts. Increased CD34-positive microvessel density was also observed in tumors from Tg mice, including those treated with GEM, suggesting PRDX4-associated vascular phenotypic changes. However, CD34 staining primarily reflects vessel density and does not directly indicate vascular functionality, perfusion efficiency, or normalization status [37]. Functional vascular assessments were not performed in this study, and therefore the observed vascular increase reflects phenotypic rather than functional angiogenic changes. Because PRDX4 is systemically overexpressed in Tg host tissues, increased tumor PRDX4 likely reflects combined tumor and stromal contributions. Despite the absence of direct vascular functional analysis, these host–tumor interactions remain biologically and translationally relevant, as they coincide with enhanced tumor growth and reduced chemotherapy responsiveness. Future studies incorporating vascular perfusion and hypoxia analyses will help clarify the relationship between PRDX4 expression, tumor vascular features, and chemoresistance.

PRDX4-associated resistance is likely multifactorial. Intrinsically, PRDX4-associated antioxidant capacity and activation of PI3K/AKT and ERK signaling may mitigate oxidative and metabolic stress induced by GEM. Extrinsically, PRDX4-associated vascular remodeling may reduce drug accessibility and sustain tumor survival. The contrast between extensive necrosis in GEM-treated WT tumors and limited necrosis in PRDX4-overexpressing tumors supports an integrated resistance phenotype.

From a translational perspective, PRDX4 may represent a clinically relevant biomarker in PDAC. Given its localization within the endoplasmic reticulum and secretory pathway, PRDX4 may have the potential to be released into the extracellular space. However, circulating PRDX4 levels were not evaluated in the present study, and no direct evidence currently supports its role as a liquid biopsy biomarker in PDAC. Therefore, this possibility should be considered hypothesis-generating, and further studies are required to determine whether serum or plasma PRDX4 could serve as a clinically useful minimally invasive biomarker. In addition, PRDX4 immunohistochemistry may be feasible in routine pathological evaluation following prospective validation. From a clinical perspective, systemic treatment strategies for advanced PDAC have evolved substantially over the past decade. Combination chemotherapy regimens such as FOLFIRINOX or GEM plus nab-paclitaxel are now recommended as first-line treatments and have demonstrated superior survival outcomes compared with GEM monotherapy [38]. In this context, our findings regarding PRDX4-associated redox adaptation and chemoresistance may have broader relevance beyond GEM-based therapy and could potentially extend to modern combination regimens, although this requires further investigation. Although direct therapeutic targeting of PRDX4 was not addressed, emerging evidence suggests that PDAC may depend on PRDX4-associated ER redox homeostasis, providing a rationale for exploring PRDX4-related vulnerabilities [39].

This study has several limitations. First, the ROC-derived cut-off value for PRDX4 expression was established and evaluated within the same cohort, and external validation in independent cohorts will be required. Second, the relatively small animal cohort reflects the exploratory nature of the in vivo experiments and requires validation in larger studies. Third, although the present study provides integrated clinical and experimental evidence supporting PRDX4-associated phenotypes in PDAC, complementary loss-of-function studies will be necessary to further clarify the endogenous requirement and mechanistic role of PRDX4. Finally, subcutaneous and transgenic tumor models may not fully recapitulate orthotopic tumor architecture and vascular functionality.

In summary, PRDX4 expression is associated with redox-related molecular alterations and aggressive tumor-associated phenotypes in PDAC. PRDX4 overexpression coincides with altered intracellular ROS levels, coordinated signaling and antioxidant protein changes, enhanced tumor cell proliferation and migration in vitro, and increased tumor growth with reduced GEM responsiveness in vivo. Clinically, elevated PRDX4 expression identifies PDAC with aggressive behavior and unfavorable prognosis. These findings support PRDX4 as a clinically relevant biomarker linked to PDAC progression and therapeutic response and provide a rationale for further investigation of PRDX4-associated redox vulnerabilities in PDAC.

## Ethics approval and consent to participate

This study was approved by the Ethics Committee of Kanazawa Medical University (Approval No I233).

This study complied with the Declaration of Helsinki. The written consent was received from all participants.

All animal experimental procedures were performed in accordance with institutional guidelines for the care and use of laboratory animals in Japan.

## Consent for publication

All authors have approved the final version of the manuscript and have given their consent for publication.

## Funding

This work was supported in part by the Grants-in-Aid for Scientific Research 20K0745K to S.Y. from the Ministry of Education, Culture, Sports, Science and Technology, Tokyo, Japan; The 2025 Kisshokai Foundation to S.Y., Ishikawa, Japan; The Foundation for Cooperative Research in Kanazawa Medical University to S.Y., Ishikawa, Japan (C2023-4); The Grant for Assist KAKEN from Kanazawa Medical University to J.H., Ishikawa, Japan (K2025-20).

## CRediT authorship contribution statement

**Yao Liu:** Conceptualization, Methodology, Investigation, Formal analysis, Writing – original draft. **Jia Han:** Methodology, Investigation, Data curation, Writing – review & editing. **Akihiro Shioya:** Validation, Investigation, Writing – review & editing. **Takeru Oyama:** Validation, Investigation, Writing – review & editing. **Xin Guo:** Supervision, Writing – review & editing. **Qian Yang:** Supervision, Resources, Writing – review & editing. **Hidetaka Uramoto:** Supervision, Resources, Writing – review & editing. **Sohsuke Yamada:** Conceptualization, Supervision, Funding acquisition, Writing – review & editing.

## Declaration of competing interest

The authors state that there are no financial, personal, or professional conflicts of interests that may hinder this work.

## Data Availability

The datasets used and/or analyzed during the current study available from the corresponding author on reasonable request.

## Appendix Supplementary materials

Image, application 1

Image, application 2
