# Supplementary material for: PRDX4 expression potentially links redox adaptation to oncogenic signaling and tumor progression in pancreatic ductal adenocarcinoma
Source: Transl Oncol. 2026 Jun 20;71:102865. doi: 10.1016/j.tranon.2026.102865 (PMC13312543; doi:10.1016/j.tranon.2026.102865)
Supplement: Supplementary file 1 [file mmc1.docx]

**Supplementary Figures.**


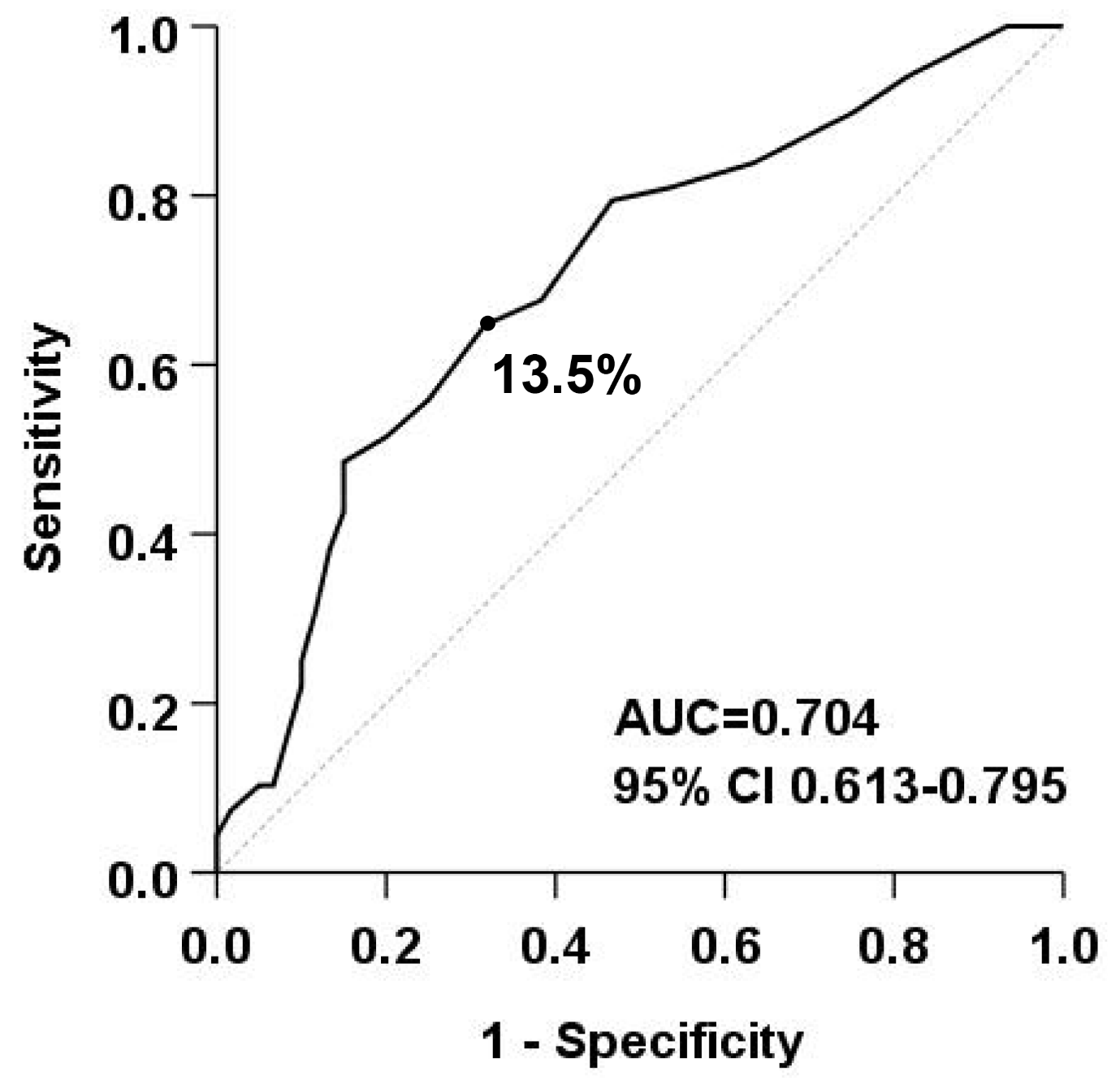


**fig. S1.** ROC curve analysis for selecting a Youden index based cut-off value of PRDX4 expression (exploratory). ROC curve showing the discriminatory performance of PRDX4 expression in PDAC. The optimal cut-off value of 13.5% was determined based on the Youden index, yielding an area under the curve (AUC) of 0.703 (95% CI: 0.616-0.791). This data driven threshold was used to dichotomize patients into high and low PRDX4 expression groups for subsequent analyses.


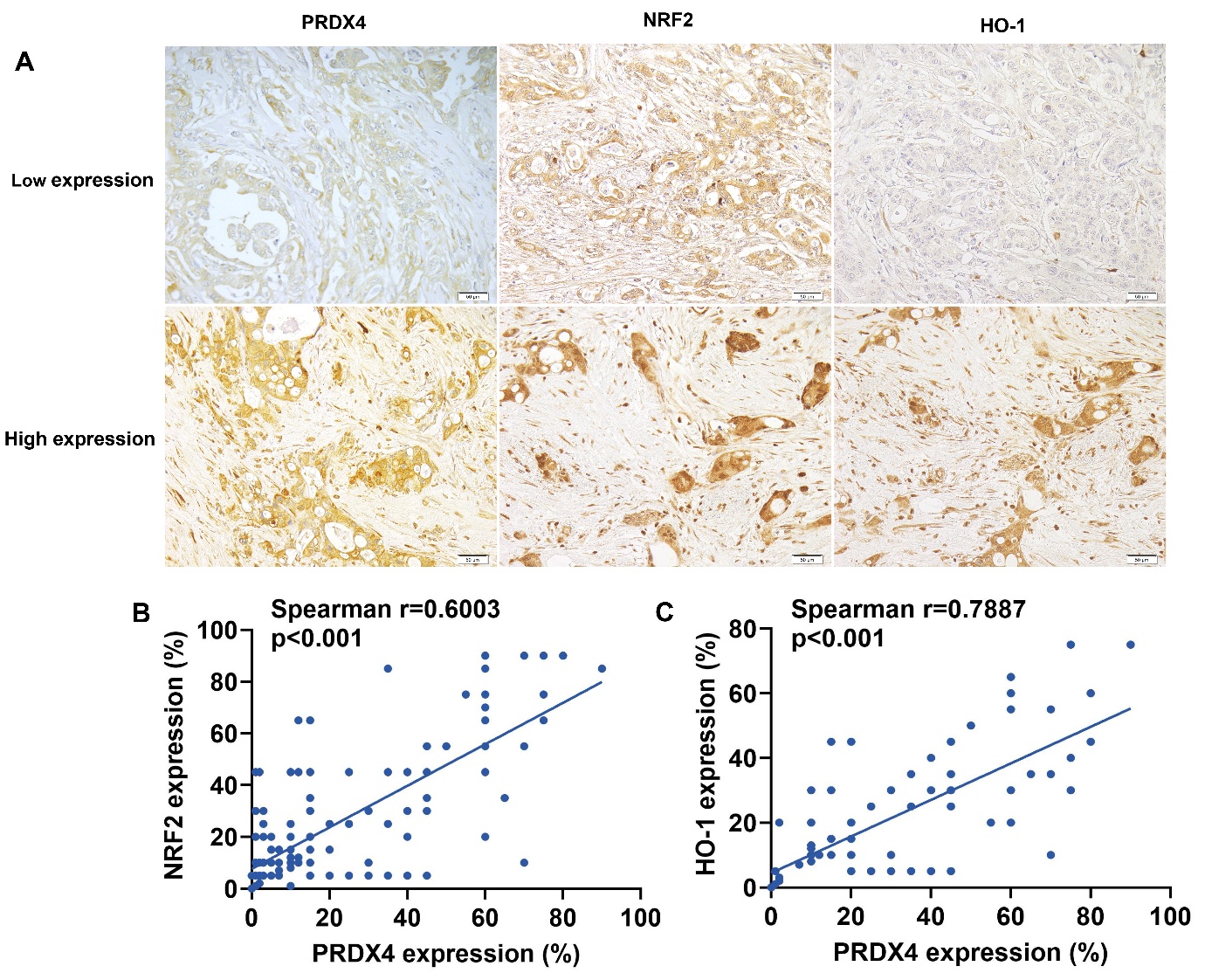


**fig. S2.** Correlation between PRDX4 expression and downstream redox-related proteins in clinical PDAC specimens. (A) Representative immunohistochemical staining showing low and high expression of PRDX4, NRF2, and HO-1 in PDAC tissues. Tumors with high PRDX4 expression exhibited increased NRF2 and HO-1 positive staining cells, whereas tumors with low PRDX4 expression showed decreased positive staining cells. (B) Scatter plot showing a positive correlation between PRDX4 and NRF2 expression (Spearman r=0.6003, *P*<0.001). (C) Scatter plot showing a positive correlation between PRDX4 and HO-1 expression (Spearman r=0.7887, *P*<0.001).


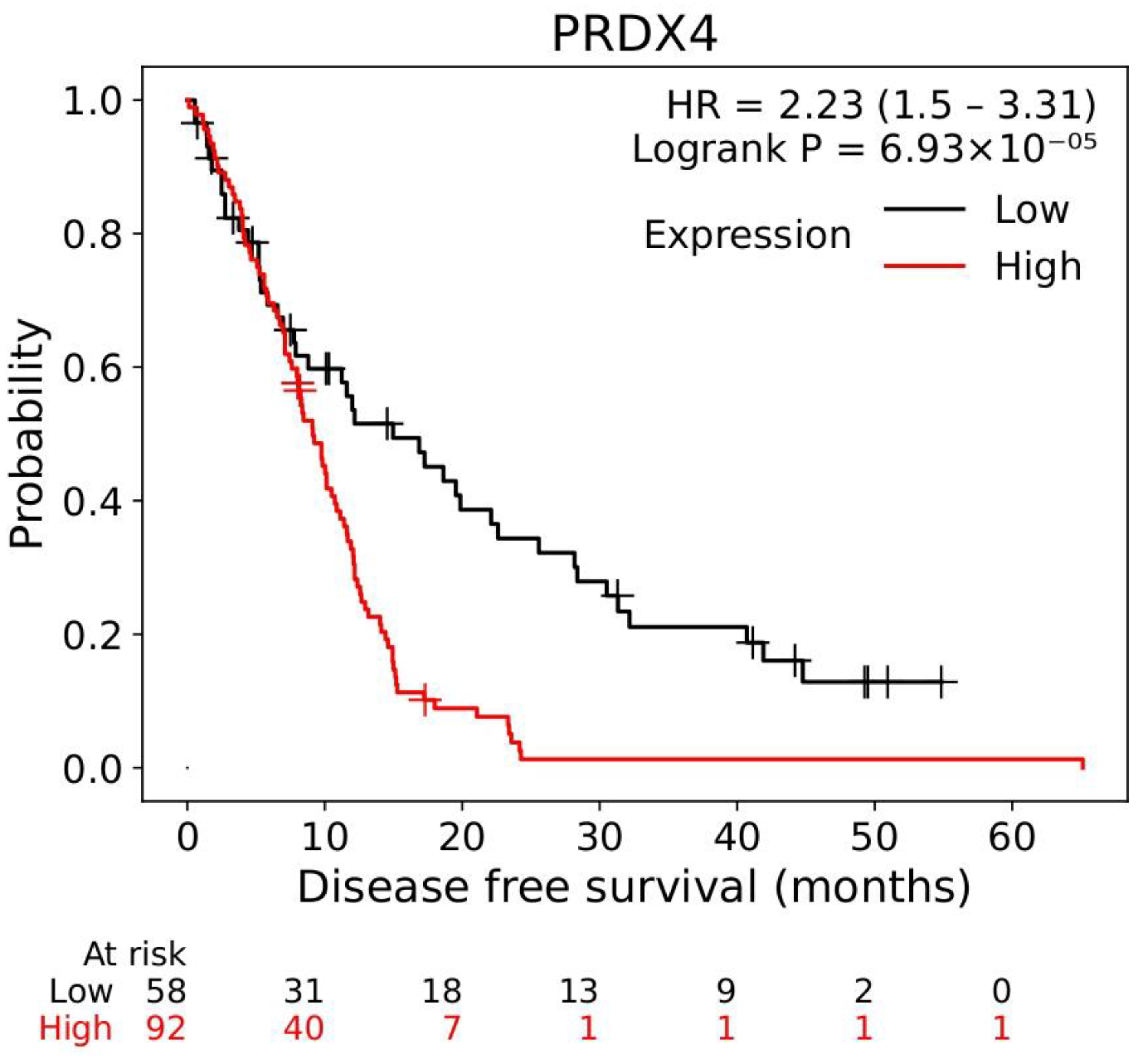


**fig. S3**. Kaplan-Meier analysis of disease-free survival (DFS) according to PRDX4 expression in pancreatic cancer. Kaplan-Meier survival curves showing DFS stratified by PRDX4 expression levels based on TCGA-derived data (Kaplan-Meier Plotter). Patients with high PRDX4 expression (red line) exhibited significantly poorer DFS compared with those with low expression (black line). Hazard ratio (HR)=2.23 (95% CI: 1.50-3.31), log-rank *P*=6.93×10^-5^. Numbers at risk are indicated below the plot.


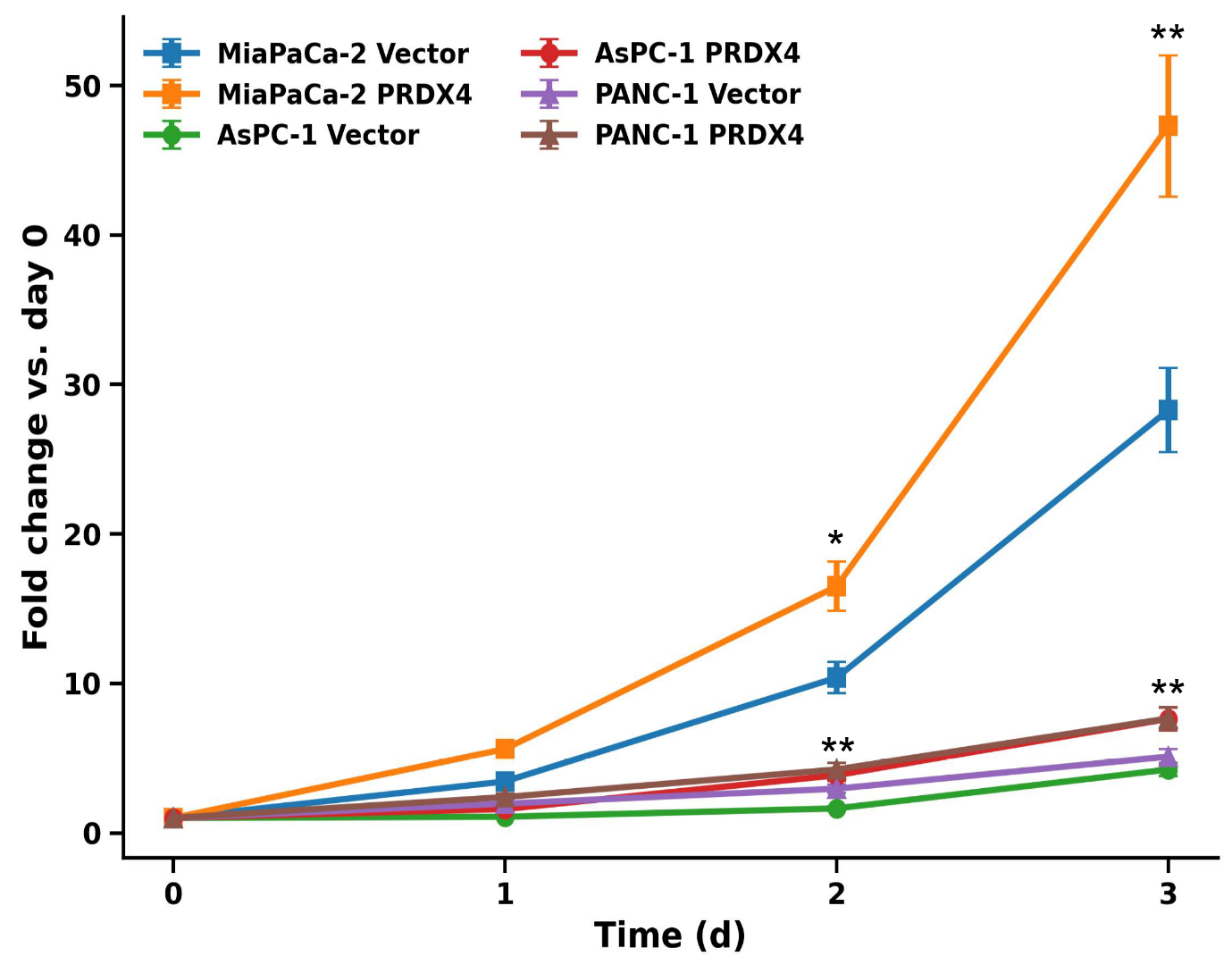


**fig. S4.** Fold-change analysis of cell proliferation following PRDX4 overexpression in PDAC cell lines. Proliferation curves are presented as fold-change relative to day 0 to facilitate comparison across cell lines with different baseline OD values. PRDX4 overexpression consistently increased relative proliferation rates in MiaPaCa-2, AsPC-1, and PANC-1 cells. The relative increase was most pronounced in MiaPaCa-2 cells, while similar trends were observed across all cell lines.


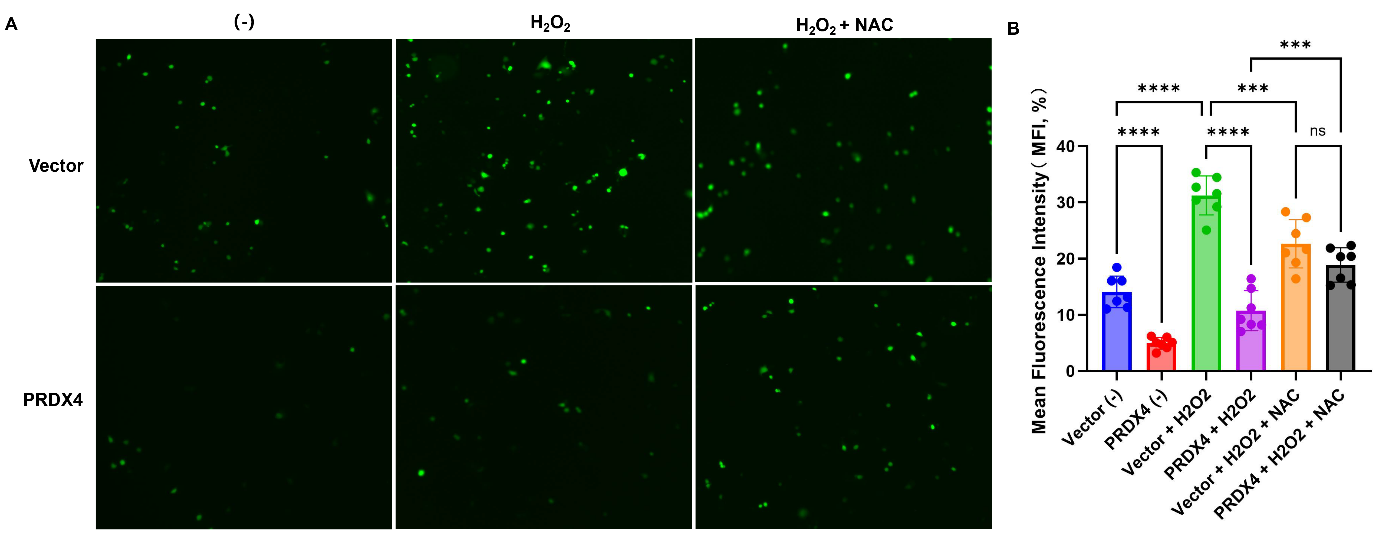


**fig. S5.** Validation of PRDX4-associated ROS modulation in MiaPaCa-2 cells. (A) Representative fluorescence images of intracellular ROS levels detected using the DCFH-DA probe in vector control and PRDX4-overexpressing MiaPaCa-2 cells under basal conditions (−), after H_2_O_2_ treatment, and after combined H_2_O_2_ and NAC treatment. Cells were treated with H_2_O_2_ (200μM, 30 min) to induce oxidative stress. For antioxidant rescue experiments, cells were pretreated with NAC for 1 h prior to H_2_O_2_ exposure. Scale bars, 200μm. (B) Quantitative analysis of intracellular ROS levels expressed as MFI. PRDX4 overexpression was associated with reduced ROS levels under basal conditions and attenuated ROS accumulation following H_2_O_2_ exposure. NAC treatment reduced ROS levels and largely diminished the differences between groups. Data are presented as mean±SD from at least three independent experiments. Statistical significance was determined using Student’s t-test or one-way ANOVA as appropriate (^***^*P*<0.001, ^****^*P*<0.0001; ^ns^*P*>0.05).


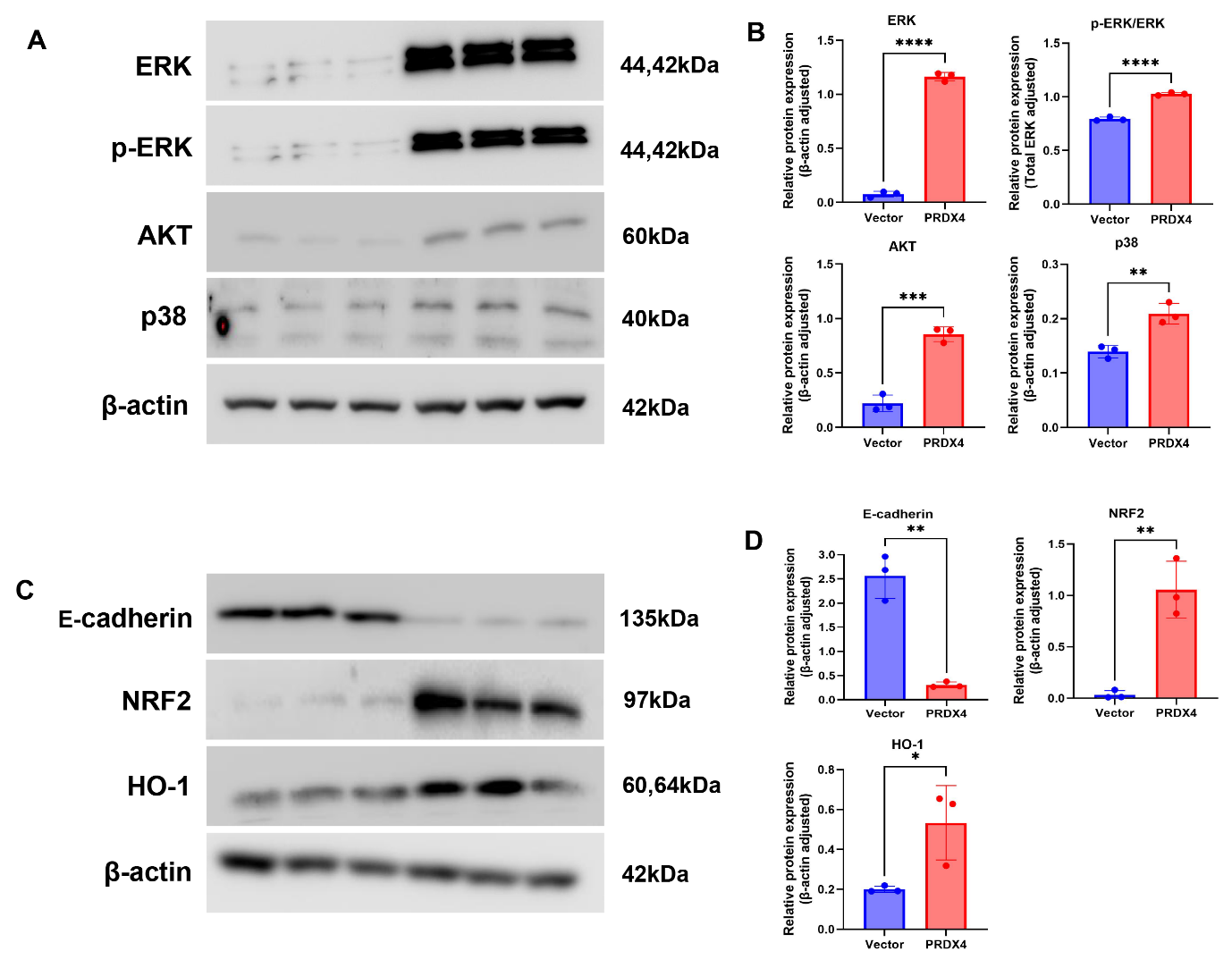


**fig. S6.** Validation of selected PRDX4-associated protein expression changes in MiaPaCa-2 cells. (A) Representative Western blot images showing the expression of ERK, phosphorylated ERK/p-ERK, AKT, and p38 in MiaPaCa-2 cells transfected with PRDX4 or vector control. (B) Quantitative analysis showing increased protein levels of ERK, p-ERK, AKT, and p38 in PRDX4-overexpressing cells compared with vector controls, normalized to β-actin (p-ERK normalized to ERK). (C) Representative Western blot images showing the expression of E-cadherin, NRF2 and HO-1 in PRDX4-overexpressing and control MiaPaCa-2 cells. (D) Quantitative analysis demonstrating reduced E-cadherin expression and increased NRF2 and HO-1 protein levels in PRDX4-overexpressing cells, normalized to β-actin. Data are presented as mean ± SD from independent experiments. Statistical significance was determined using unpaired t-tests (^*^*P*<0.05, ^**^*P*<0.01, ^***^*P*<0.001, ^****^*P*<0.0001).
