# Supplementary material for: PRDX4 expression potentially links redox adaptation to oncogenic signaling and tumor progression in pancreatic ductal adenocarcinoma
Source: Transl Oncol. 2026 Jun 20;71:102865. doi: 10.1016/j.tranon.2026.102865 (PMC13312543; doi:10.1016/j.tranon.2026.102865)
Supplement: Supplementary file 2 [file mmc2.docx]

**Table S1. Sensitivity analyses of PRDX4 expression using alternative cut-off strategies (median- and tertile-based thresholds) for DSS in patients with PDAC**

Cox proportional hazards models were adjusted for the same covariates as in the primary multivariable model. For tertile-based analysis, the highest tertile was compared with the lowest tertile.

| **Cut-off strategy** | **Group comparison** | **Outcome** | **HR (95% CI)** | **p value** |
| --- | --- | --- | --- | --- |
| Median cut-off | High vs Low | 3-year DSS | 2.011 (1.532-2.608) | 0.027 |
|  |  | 5-year DSS | 2.182 (1.476-3.225) | 0.031 |
| Tertile cut-off | High vs Low | 3-year DSS | 2.671 (1.925-3.743) | 0.037 |
|  |  | 5-year DSS | 2.655 (1.605-4.156) | 0.041 |

**Table S2. Interobserver agreement for PRDX4 expression**

| Items | Pathologist Pair | Cohen's Kappa (κ) | 95% CI | Agreement Strength |
| --- | --- | --- | --- | --- |
| **PRDX4** | **Y.L. vs J.H.** | 0.824 | 0.768–0.879 | Almost Perfect |
|  | **Y.L. vs S.Y.** | 0.781 | 0.715–0.846 | Substantial |
|  | **J.H. vs S.Y.** | 0.693 | 0.618–0.758 | Substantial |
|  | **Overall (Fleiss’ κ)** | 0.802 | 0.618–0.758 | Almost Perfect |

Interpretation: Agreement strength was interpreted as follows: <0.20, slight; 0.21-0.40, fair; 0.41-0.60, moderate; 0.61-0.80, substantial; 0.81-1.00, almost perfect.

**Table S3. Model diagnostics for multivariable Cox proportional hazards model in the excluding cohort**

Tests of the proportional hazards (PH) assumption and multicollinearity for the primary models. The PH assumption was evaluated using Schoenfeld residuals; *P*>0.1 indicates no violation. Multicollinearity was assessed by variance inflation factor (VIF); VIF < 2.0 was considered acceptable.

| **Variables** | **Collinearity Tolerance** | **VIF** | **Outcome** | **Global PH test (P)** |
| --- | --- | --- | --- | --- |
| Location | 0.956 | 1.046 | 3-year DSS | 0.287 |
| Tumor size | 0.876 | 1.141 |  |  |
| Differentiation | 0.977 | 1.023 |  |  |
| Vascular invasion | 0.749 | 1.335 |  |  |
| TNM stage | 0.695 | 1.439 |  |  |
| PRDX4 expression | 0.813 | 1.230 |  |  |
| Location | 0.964 | 1.037 | 5-year DSS | 0.311 |
| Differentiation | 0.986 | 1.014 |  |  |
| Lymphatic vessel invasion | 0.665 | 1.770 |  |  |
| Vascular invasion | 0.683 | 1.513 |  |  |
| TNM stage | 0.765 | 1.307 |  |  |
| PRDX4 expression | 0.813 | 1.230 |  |  |

**Table S4. Model diagnostics for multivariable Cox proportional hazards model in the entire cohort**

Tests of the proportional hazards (PH) assumption and multicollinearity for the primary models. The PH assumption was evaluated using Schoenfeld residuals; *P*>0.1 indicates no violation. Multicollinearity was assessed by variance inflation factor (VIF); VIF < 2.0 was considered acceptable.

| **Variables** | **Collinearity Tolerance** | **VIF** | **Outcome** | **Global PH test (P)** |
| --- | --- | --- | --- | --- |
| Tumor size | 0.893 | 1.120 | 3-year DSS | 0.352 |
| Differentiation | 0.980 | 1.020 |  |  |
| Vascular invasion | 0.762 | 1.313 |  |  |
| TNM stage | 0.740 | 1.351 |  |  |
| PRDX4 expression | 0.799 | 1.251 |  |  |
| Differentiation | 0.997 | 1.003 | 5-year DSS | 0.269 |
| TNM stage | 0.877 | 1.140 |  |  |
| PRDX4 expression | 0.875 | 1.143 |  |  |

**Table S5. Sensitivity analysis using a fixed multivariable Cox model with prespecified covariates in the pM1-excluded cohort**

Fixed covariates included in both models: age, gender, tumor differentiation, TNM stage, and PRDX4 expression.Fixed covariates included in both models: age, gender, tumor differentiation, TNM stage, and PRDX4 expression.

| **Variables** | **3-year DSS** | | | **5-year DSS** | | |
| --- | --- | --- | --- | --- | --- | --- |
|  | **HR** | **95% CI** | **p Value** | **HR** | **95% CI** | **p Value** |
| Age | 1.591 | 0.252-2.387 | 0.227 | 1.459 | 0.208-3.013 | 0.154 |
| Gender | 1.248 | 0.732-2.127 | 0.416 | 1.359 | 0.844-2.188 | 0.207 |
| Differentiation | 1.601 | 1.066-2.404 | 0.023 | 1.401 | 0.963-2.038 | 0.078 |
| TNM stage | 2.423 | 1.243-4.724 | 0.009 | 1.915 | 1.107-3.313 | 0.020 |
| PRDX4 expression | 1.859 | 1.079-3.202 | 0.025 | 1.478 | 1.033-3.391 | 0.023 |

Note: A fixed multivariable Cox proportional hazards model was used for both 3-year and 5-year DSS to reduce model selection instability and improve comparability across endpoints. Covariates were prespecified based on clinical relevance rather than selected solely according to univariate *P* values.
